# ﻿Diversity of *Orbiniella* (Orbiniidae, Annelida) in the North Atlantic and the Arctic

**DOI:** 10.3897/zookeys.1205.120300

**Published:** 2024-06-20

**Authors:** Miguel A. Meca, Jon Anders Kongsrud, Katrine Kongshavn, Tom Alvestad, Karin Meißner, Nataliya Budaeva

**Affiliations:** 1 Department of Natural History, University Museum of Bergen, University of Bergen, Bergen, Norway University of Bergen Bergen Norway; 2 Senckenberg Forschungsinstitute und Naturmuseun, German Centre for Marine Biodiversity Research, Hamburg, Germany Senckenberg Forschungsinstitute und Naturmuseun, German Centre for Marine Biodiversity Research Hamburg Germany

**Keywords:** Greenland-Iceland-Scotland Ridge, integrative taxonomy, molecular marker, new species, Nordic seas, SEM, species complex, species delimitation

## Abstract

In this work, the diversity of the genus *Orbiniella* in the Nordic Seas and the North Atlantic waters south of Iceland is studied based on the analyses of molecular markers (mitochondrial COI, 16S rDNA and nuclear ITS2) and morphological characters. Our results showed the presence of at least five genetic lineages in the studied material which could also be morphologically identified by their segmental annulation patterns, the number and the shape of acicular spines, and the length and the shape of pygidial lobes. The species name *Orbiniellapetersenae* is assigned to one of the lineages restricting its geographical and vertical distribution to the deep-sea areas north of Iceland and Jan Mayen, and three lineages are described as new species (i.e., *Orbiniellagriegi* Meca & Budaeva, **sp. nov.**, *Orbiniellamayhemi* Meca & Budaeva, **sp. nov.**, and *Orbiniellaparapari* Meca & Budaeva, **sp. nov.**) elevating the number of known species in the genus to 25. Three deep-sea species of *Orbiniella* in our study are reported only north of the Greenland-Iceland-Scotland Ridge, one deep-sea species found south of the ridge. A single shallow-water species is distributed along the ridge and on the Norwegian shelf.

## ﻿Introduction

The genus *Orbiniella* is the fifth most diverse genus in Orbiniidae with 22 valid species (Blake 2021). Most species (i.e., 13 of 22) occur in deep-water habitats exceeding 1000 m, with some of them inhabiting hypoxic biotopes, such as hydrothermal vents (e.g., *Orbiniellahobsonae* Blake & Hilbig, 1990) or the Clarion-Clipperton Fracture Zone in the abyssal Pacific Ocean (e.g., *Orbiniellaabyssalis* Blake, 2020). Other *Orbiniella* are known from shallow waters, occurring in the soft bottoms from the intertidal to the continental shelf.

The genus is characterised by small size (up to 12 mm long in the largest species, *Orbiniellaandeepia* Narayanaswamy & Blake, 2005), having a rounded prostomium, two achaetous segments followed by chaetigers without branchiae and, sometimes, also without parapodial lobes. The chaetae are represented by the crenulated capillary chaetae and also by the simple acicular spines. After the first described species of the genus, *Orbiniellaminuta* Day, 1954, the number of species was increasing slowly with 11 species known until recently. The drastic increase in *Orbiniella* species number happened in the last seven years with 12 more species described (Blake 2017, 2020, 2021; Georgieva et al. 2023), with most coming from deep-sea habitats.

The only *Orbiniella* species reported from the NE Atlantic until now was *Orbiniellapetersenae* Parapar, Moreira & Helgason, 2015. The species was described based on formalin-fixed material from Icelandic waters. Most of the specimens (including the holotype) were collected on the continental slope (1490 m to 1915 m depth) northeast of Iceland, in cold Arctic deep waters (-0.7 °C to -0.8 °C), whilst the other specimens were found on the shelf and upper slope (133 m to 1007 m depth) southwest of Iceland, in the warmer north-eastern Atlantic waters (4.8 °C to 7.4 °C). Despite limited geographical distribution, the reported depth and temperature ranges of *O.petersenae* are rather wide suggesting a possibility of a species complex. Numerous cases of cryptic speciation in the North Atlantic annelids were reported in the last decades based on the analyses of the combination of morphological and molecular data (e.g., Nygren and Pleijel 2011; Bogantes et al. 2018; Nygren et al. 2018; Grosse et al. 2020). The availability of the large material of *O.petersenae* recently collected during several Norwegian and German sampling programs allowed investigating genetic and morphological diversity of *Orbiniella* from various localities in the Nordic Seas (i.e., the Norwegian, Greenland, and the Iceland seas) and the adjacent waters of the North Atlantic.

The aim of the present study was to analyse morphological variation, genetic divergence, and phylogenetic relationships among several populations of *Orbiniella* inhabiting different depths and water masses in the NE Atlantic and the Nordic seas. As a result, five species were found in the studied material with the species name *O.petersenae* restricted to a particular molecular clade inhabiting bathyal depths north of Iceland and near Jan Mayen. Three of the other genetic lineages are herein described as new species.

## ﻿Material and methods

### ﻿Sampling

Specimens were collected between 1986 and 2016 during several expeditions and monitoring programs in the Nordic Seas and the NE Atlantic Ocean organised by the Department of Biological Sciences and the Centre for Deep Sea Research (University of Bergen, Norway), MAREANO (Marine Area database for Norwegian waters, Norway), and the IceAGE (Icelandic marine Animals: Genetics and Ecology, Germany) expedition organised by the “Deutsches Zentrum für Marine Biodiversitätsforschung” (DZMB), Hamburg. Some of the specimens were preserved directly in 96% ethanol for molecular analysis, whilst others were first fixed in 4% formalin and later stored in 70% ethanol (Suppl. material 1: table S1).

We analysed 964 specimens from 62 collecting sites, in the depth range 65–3892 m. The specimens are deposited in the Invertebrate collection of the University Museum of Bergen, University of Bergen, Norway (**ZMBN**) and in the Senckenberg Museum of Frankfurt, Germany (**SMF**). In addition to the collected material, we also studied the holotype and 197 paratypes of *O.petersenae* sensu lato from the collections of the Icelandic Institute of Natural History, Reykjavik, Iceland (**IINH**) (see Parapar et al. 2015 and Suppl. material 1: table S1). Species distribution maps were generated in QGIS 3.28.1.

### ﻿Morphological characterisation

Specimens were studied under a stereomicroscope and a compound microscope using temporary slides of whole specimens mounted in 96% or 70% ethanol. For a better visualisation of the morphological characters, Scanning Electron Microscopy (SEM) and methylene blue stain diluted in distilled water were applied to the individuals. For SEM, specimens were dehydrated in a graded ethanol series, critical-point dried, sputter coated with gold/palladium alloy and photographed with a ZEISS Supra 55VP scanning electron microscope at the Electron Microscopy Laboratory (ELMILAB), University of Bergen. SEM photos were edited and combined to plates using Adobe Photoshop 2021 22.3.1. Aqueous solution of methylene blue was used to add contrast to external structures, such as parapodia or pygidium and to highlight segmental borders.

Nine morphological characters used in the previous studies on *Orbiniella* (Parapar et al. 2015; Blake 2020) were assessed in the studied specimens: (1) the shape of prostomium; (2) the relative length of peristomial segments; (3) the segmental annulation pattern along the body; (4) the shape of parapodia throughout the whole body; (5) the shape and length of notopodia; (6) the number and length of capillaries along the body; (7) the number and shape of the acicular spines; (8) the number of pre-pygidial segments, and (9) the length and shape of anal lobes.

The segments in *Orbiniella* species show secondary annulation with wide and narrow annuli interchanging each other in a particular pattern. Each segment can consist of one to four annuli, i.e. being uniannulate, biannulate, triannulate, or quadriannulate. Although the annulation pattern can be clearly seen on SEM images and in methylene blue stained specimens, the borders between the segments are often difficult to identify. However, the wide annuli are always associated with parapodia, and the narrow annuli are always located between the parapodia. Parapar et al. (2015) and Blake (2017, 2020, 2021) considered segmental annulation being an informative character in species identification. In the present study, we describe the annulation patters as the number of narrow rings between the parapodia.

### ﻿DNA Extraction, PCR amplification, and sequencing

We used two mitochondrial (COI and 16S rRNA) and one nuclear (ITS2 with a flanking region of 28S) marker. DNA was extracted using QuickExtract^TM^ DNA Extraction Solution (Epicentre). A small piece of tissue, usually three or four segments, was placed into 70 μl QuickExtract^TM^ solution, and incubated at 65 °C for 45 min followed by 2 min at 95 °C in a dry block thermostat. The sets of primers and amplification protocols used for each marker are summarised in Suppl. material 1: table S2. The total volume of each PCR reaction was 25 μl containing: 17.35 μl of nuclease-free water, 2.5 μl of Buffer (10X), 2 μl of nucleotide mix (2.5 mM each dNTP), 1 μl of each primer (10 µM), 0.15 μl of TaKaRa Taq DNA polymerase (Clontech, concentration of 5 U/µl,), 1 μl of template DNA. Amplified PCR products were analysed by electrophoresis on a 1% agarose gel stained with GelRed Nucleic Acid Stain and then sent to Macrogen Inc. facilities (Amsterdam, the Netherlands) for purification and bidirectional sequencing. Consensus sequences were generated and edited in Geneious Prime 2020.1.2 (Biomatters Ltd., Auckland, New Zealand) (Kearse et al. 2012).

### ﻿Phylogenetic analyses and genetic distances

Molecular data were obtained for 54 specimens of *Orbiniella*. COI and 16S sequences of *Orbiniellaplumisetosa* Buzhinskaya, 1993 (Bleidorn 2005; Bleidorn et al. 2009) as well as four 16S sequences of *Orbiniella* sp. 49 PB and *Orbiniella* sp. 279 PB (Bonifácio et al. 2020) were obtained from GenBank. Sequences of *Nainerisquadricuspida* (Fabricius, 1780) and *Phylonorvegicus* (M. Sars in G. O. Sars, 1872) (Bleidorn et al. 2009) from GenBank were used as outgroups in COI, 16S and in the combined analysis (Suppl. material 1: table S1). ITS2 sequences were analysed without outgroups since the ITS2 sequences of other orbiniids available in GenBank were too dissimilar to align with our data. The dataset of each marker was aligned individually using MAFFT online service 7.475 (Katoh et al. 2019) under the L-INS-i strategy. Alignments were concatenated with Geneious Prime. Best-fit models for each partition were selected using the Akaike Information Criterion with small sample correction (AICc) (Sugiura 1978) in Partition Finder 2.1.1 (Lanfear et al. 2017). We applied the Symmetrical model with an estimated proportion of invariant sites and gamma distributed across sites (GTR+I+G) for the 16S, ITS2 and the first and second codon positions of COI, and the Hasegawa-Kishino-Yano model gamma distributed across sites (HKY+G) for the third codon position of COI. Phylogenetic analyses were done for individual markers and for the concatenated matrix composed of three markers in CIPRES Science Gateway 3.3 (Miller et al. 2012). Maximum Likelihood (ML) analyses were conducted in IQ-TREE 2.0.5 (Nguyen et al. 2015) with 1000 ultrafast bootstrap replicates. Bayesian Inference (BI) analyses were done in MrBayes 3.2.7 (Ronquist et al. 2012) with two independent runs (each performed for eight Markov Chain Monte Carlo simulations) for 40 million generations for the individual data sets and for 100 million for the combined data set, sampled every 1000 generations and 25% of the initial trees discarded as burn-in. We considered convergence of runs (Average Standard Deviation of Split Frequencies (ASDSF) < 0.03) and effective sample size of parameters (ESS > 200) calculated in Tracer 1.7.1 (Rambaut et al. 2018) to evaluate the runs and accept results of the analyses. The resulting ML and BI trees were visualised in Figtree 1.4.4 (http://tree.bio.ed.ac.uk/software/figtree/). The concatenated BI tree was edited in CorelDRAW X7, whilst the rest of the trees were edited with Inskape 1.1 (https://inkscape.org/). For the three genes individually, uncorrected p-distances with gaps treated as pairwise deletion were calculated in MEGAX 10.2.4 (Kumar et al. 2018).

### ﻿Species delimitation

To delineate putative species in our data sets, the Poisson Tree Processes model (PTP) (Zhang et al. 2013) and the Assemble Species by Automatic Partitioning (ASAP) (Puillandre et al. 2021) were used for individual markers. PTP was inferred through its webserver (https://species.h-its.org), using the obtained BI rooted trees for COI, 16S and unrooted tree for ITS2, 100000 generations and default settings. Outgroups were removed from the COI and 16S trees. The convergence of MCMC runs was checked in the maximum likelihood plot generated by the software. The ASAP was applied through its webserver (https://bioinfo.mnhn.fr/abi/public/asap/asapweb.html) using alignment files and a p-distance model.

## ﻿Results

### ﻿Phylogenetic analyses and genetic distances

The COI alignment was 662 bp long and comprised 38 sequences, 419 variables sites, and 404 parsimony-informative sites. The 16S alignment was 525 bp long and contained 51 sequences, 392 variables sites and 358 parsimony-informative sites, and the ITS2 alignment was 906 bp long and included 29 sequences, 478 variable sites, and 464 parsimony-informative sites. The final combined dataset consisted of 2093 bp and 61 sequences.

**Figure 1.**
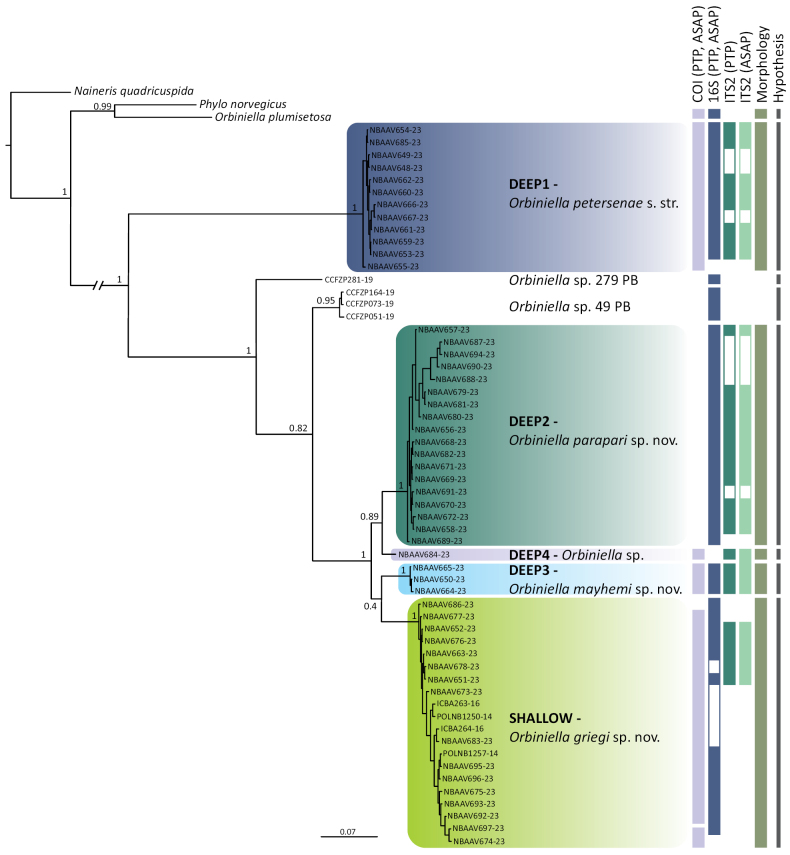
Bayesian inference (BI) based on the concatenated dataset of COI, 16S and ITS2. Bayesian posterior probabilities are shown on the nodes. Capital letters correspond with the clades discussed in the text. Species delimitation results inferred by DNA-based methods and morphology are indicated right to the BI tree along with the final species delimitation hypothesis. White bars indicate missing data.

Both ML and BI trees for the combined dataset showed similar topologies, with specimens from the NE Atlantic and the Nordic seas grouping into four well-supported clades: Deep 1, Deep 2, Deep 3, and Shallow (BS between 95 and 100% and PP of 1) (Fig. 1, Suppl. material 2: fig. S1). The clade Deep 4 was represented by a single specimen. *Orbiniella* sp. 49 PB was sister to the clade combining all the NE Atlantic/Nordic specimens except Deep 1 (BS = 94%; PP = 1). *Orbiniella* sp. 279 PB was sister to the clade combining *Orbiniella* sp. 49 PB, Shallow, Deep 2, Deep 3, and Deep 4 (BS = 60%; PP = 0.82). The only difference between the ML and BI trees for the combined matrix was the position of the Deep 3 clade. In BI analysis, Deep 3 formed a poorly supported clade with Shallow (PP = 0.4; Fig. 1). In ML analysis, Deep 3 was sister to the poorly supported group including the clades Shallow, Deep 2, and Deep 4 (BS = 56%; Suppl. material 2: fig. S1). The topology of both ML and BI trees of the individual markers was the same for the three genes (Suppl. material 2: figs S2–S4), except for the BI of 16S in which Deep 3 was sister to the clade comprising Shallow and Deep 2 (PP = 0.5; Suppl. material 2: fig. S5). *Orbiniellaplumisetosa* never fell within the ingroup (i.e., NE Atlantic and Nordic species, *Orbiniella* sp. 49 PB and *Orbiniella* sp. 279 PB), being sister with low support (BS < 75%; PP < 0.7) to *Phylonorvegicus* in all phylogenetic analyses except in the concatenated BI, in which was highly supported (PP = 0.99; Fig. 1).

The uncorrected p-distances within and between the NE Atlantic/Nordic clades and other *Orbiniella* sequences are summarised in Suppl. material 1: table S3. Apart from *O.plumisetosa*, *Orbiniella* sp. 49 PB, and *Orbiniella* sp. 279 PB, the within-clade average p-distances varied from 0 to 0.56%, and the between clade average p-distances varied from 17.86 to 29.68% in COI; 6.59–22.24% in 16S, and 2.05–30.6% in ITS2.

### ﻿Species delimitation

Results of PTP based on COI returned six putative species with a support > 0.99 corresponding to four of the five NE Atlantic/Nordic clades, *Orbiniellaplumisetosa*, and the specimens NBAAV674-23 and NBAAV697-23 delimited as a separate species (Suppl. material 2: File S1. A). The sequences of the Deep 2 clade, *Orbiniella* sp. 279 PB, and *Orbiniella* sp. 49 PB were absent in the COI dataset. The PTP for 16S resulted in seven putative species with a support > 0.97 belonging to four of the five NE Atlantic/Nordic clades, *Orbiniella* sp. 279 PB, *Orbiniella* sp. 49 PB, and *O.plumisetosa* (Suppl. material 2: File S1. B). The sequences of the Deep 4 clade were absent in the 16S dataset. The PTP for ITS2 returned five putative species with a support > 0.99 corresponding to the five NE Atlantic/Nordic clades (Suppl. material 2: File S1. C).

The best ASAP partition (i.e., showing the lowest score) based on COI returned six groups corresponding to four of the five NE Atlantic/Nordic clades except Deep 2 since it was missing in the COI dataset (File S2. A). *Orbiniellaplumisetosa*, and the specimens NBAAV674-23 and NBAAV697-23 were delimited as two separate species. The best ASAP partition for 16S included seven groups belonging to four of the five NE Atlantic/Nordic clades except Deep 4 since it was missing in the 16S dataset, *Orbiniella* sp. 279 PB, *Orbiniella* sp. 49 PB, and *O.plumisetosa* (File S2. B). The best ASAP partition for ITS2 gave four groups corresponding to the three of the NE Atlantic/Nordic clades, and Deep 3 together with Deep 4 merging in the same group (Suppl. material 2: File S2. C).

Combining the results of the phylogenetic analyses, genetic distances, and species delimitation, we consider five distinct species of *Orbiniella* present in the NE Atlantic/Nordic region (i.e., corresponding to the clades Deep 1, Deep 2, Deep 3, Deep 4, and Shallow).

### ﻿Morphological data

For the morphological analysis, all DNA vouchers belonging to a single genetic lineage were analysed in search of unambiguous characters for species delimitation. Once each species was morphologically defined, we also checked for intraspecific variation within the species using additional material.

From the nine selected characters, three were consistent and taxonomically informative among the species: the number of narrow annuli between parapodia and their pattern in the anterior body region, the number and shape of the acicular spines, and the length and shape of the anal lobes. The shape of the prostomium, peristomium and notopodia showed a great intraspecific variation within Deep 1 and Deep 2 clades. For Deep 3 and Shallow clades these characters were not variable among the specimens analysed. For the specimens from Deep 2 clade collected from the Loki’s Castle hydrothermal vent site, these characters showed less variation than from the other localities. The number of capillaries in each parapodial ramus, the length of capillary chaetae, and the position of acicular spines varied in the specimens from all genetic lineages (see species descriptions below). The number of capillary and acicular spines was variable throughout the body even within a single specimen. The length of capillaries varied from being equal to body width to longer than body width. Acicular spines were disposed as an anterior row in the upper side of the capillary bundle, as a posterior row in the lower side of the bundle or mixed with capillaries. If only a single spine was present per ramus, they were placed in vis-à-vis position in the parapodium (i.e., one spine in the notopodium directed towards the other spine in the neuropodium).

**Figure 2.**
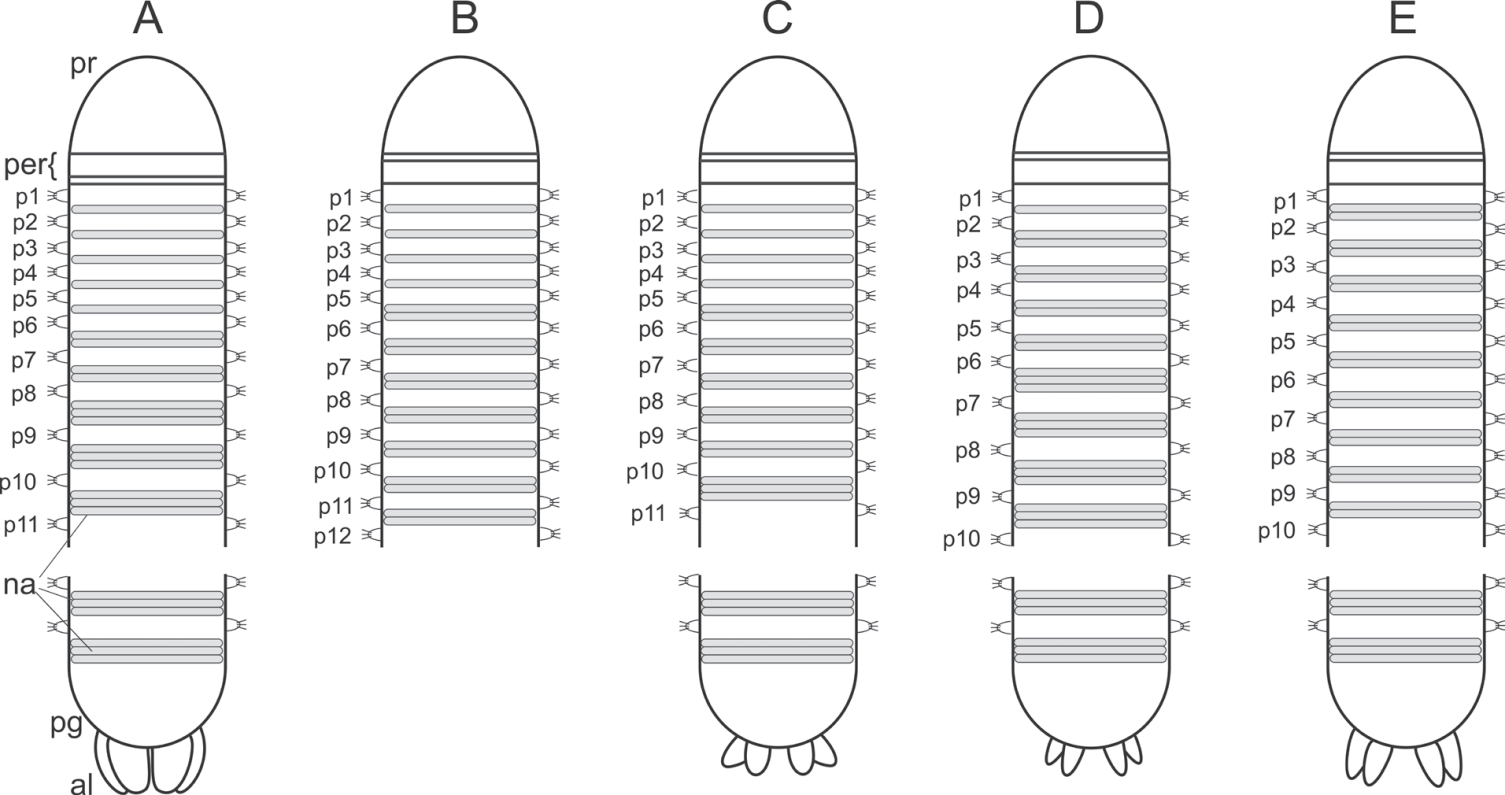
Schematic illustrations of the segmental annulation pattern and the shape of pygidial lobes in the studied *Orbiniella* species. Exact borders between the body segments are hard to define, the number of narrow annuli between parapodia that are used in species diagnoses are shown in grey colour **A***Orbiniellagriegi* Meca & Budaeva, sp. nov. (Shallow) **B***Orbiniellamayhemi* Meca & Budaeva, sp. nov. (Deep 3) **C***Orbiniellaparapari* Meca & Budaeva, sp. nov. (Deep 2) **D***Orbiniellapetersenae* sensu stricto (Deep 1) **E***Orbiniella* sp. (Deep 4). Abbreviations: al: anal lobes; na: narrow annuli; per: peristomium; pg: pygidium; pr: prostomium; p1–p12: parapodia number in the anterior body region.

When using light microscopy, the segmental annulation was not always clear; however, they become more visible after applying methylene blue staining. Five different patterns in the number of narrow annuli between parapodia were recorded, one per species. The different segmental annulation patterns are illustrated in Fig. 2 and are explained as follows: **Deep 1**, one narrow annulus between parapodium 1 and 2, two narrow annuli between parapodia from parapodium 2 until 5–6, and three narrow annuli between parapodia from parapodium 5–6 until pygidium; **Deep 2**, one narrow annulus between parapodia from parapodium 1 until 5–6, two narrow annuli between parapodia from parapodium 5–6 until 10–14, and three narrow annuli between parapodia from parapodium 10–14 until pygidium; **Deep 3**, one narrow annulus between parapodia from parapodium 1 until 5–6 and two narrow annuli between parapodia from parapodium 5–6 until end of available fragment of the most complete specimen; **Deep 4**, two narrow annuli between parapodia from parapodium 1 until posterior body and three narrow annuli between parapodia in posterior-most body. **Shallow**, one narrow annulus between parapodia from parapodium 1 until 6, two narrow annuli between parapodia from parapodium 6 until 8, and three narrow annuli between parapodia from parapodium 8 until pygidium.

### ﻿Systematic account

The integrative analysis of the NE Atlantic/Nordic *Orbiniella* material resulted in assigning the species name *Orbiniellapetersenae* to Deep 1 clade and in the description of Deep 2 clade as *Orbiniellaparapari* Meca & Budaeva, sp. nov., Deep 3 clade as *Orbiniellamayhemi* Meca & Budaeva, sp. nov. and Shallow clade as *Orbiniellagriegi* Meca & Budaeva, sp. nov. Deep 4 clade was represented by three incomplete specimens with genetic data available for only a single specimen. A similar morphotype to Deep 4 clade was found in formalin preserved specimens from the Norwegian and the Iceland Seas. We provide a detailed description of the morphology of the Deep 4 clade including morphologically similar formalin-fixed specimens, but do not name the species in the present study due to shortage of material.

**Orbiniidae Hartman, 1942**

#### ﻿ Orbiniella

Taxon classificationAnimaliaOrbiniidaOrbiniidae

Day, 1954

D63DC385-6406-532E-9F04-26957C160FA9

Falklandiella Hartman, 1967: 109. FideParapar et al. 2015: 333.
Orbiniella – Parapar et al. 2015: 333; Blake 2017: 109; Blake 2020: 38; Blake 2021: 99.

##### Type species.

*Orbiniellaminuta* Day, 1954, by monotypy.

##### Diagnosis

(emended from Blake 2021). Body usually elongated, not divided into thorax and abdomen. In some species segmental size can change gradually between anterior and posterior body. Prostomium broad or elongate with rounded anterior margin. One pair of nuchal organs usually present, sometimes pigmented. Eyes present or absent. Peristomium usually bearing two segments. Secondary annulation present with segments being uniannulate, biannulate, triannulate, or quadriannulate. Parapodia biramous with only simple postchaetal lobes, or these entirely absent. Capillary noto- and neurochaetae always crenulated or weakly crenulated with pointed tips; prominent acicular spines present in neuropodia and, usually, also in notopodia; furcate chaetae absent. Branchiae absent. Pygidium with two or four anal lobes, with or without cirri.

##### Remarks.

Blake reviewed *Orbiniella* in his monographs from 2017, 2020 and 2021, updating its generic diagnosis and the checklist of species. He reported segmental annulation pattern as a key character to separate *Orbiniella* species (Blake 2020), but this character was not included in the generic diagnosis. Similarly, Blake (2020) also reported and illustrated the anal lobes and cirri in the species descriptions without including them in the diagnosis. We consider these two characters, in combination with other characters discussed below, of high diagnostic value to separate *Orbiniella* from other orbiniid genera and here we include them in the generic diagnosis of *Orbiniella*.

The main diagnostic characters to identify *Orbiniella* species are absence of branchiae along with presence of acicular spines through the whole body, secondary annulation, and pygidium with or without anal cirri. *Microrbinialinea* Hartman, 1965, and some species of *Questa* also do not have branchiae; however, the former can be separated from *Orbiniella* by the presence of unusual long and serrated spines and the latter by the presence of bi- or tridentate crotchets. The other orbiniid having acicular spines through the whole body is *Methanoariciadendrobranchiata* Blake, 2000. However, this species has very specialised morphology (i.e., elongate and narrow prostomium, long branched branchiae, long and cirriform parapodial lobes and pygidium with many anal cirri) and is easily distinguished. Secondary annulation has been reported also in some species of *Questa* and in *Microrbinialinea* Hartman, 1965, but they can be separated from *Orbiniella* by their unique chaetae mentioned before. Most orbiniids bear two or four (rarely many) anal cirri in the pygidium, and only some *Orbiniella* and *Questa* show no anal cirri, being a unique feature of both genera.

#### ﻿ Orbiniella griegi

Taxon classificationAnimaliaOrbiniidaOrbiniidae

Meca & Budaeva, sp. nov.

76B4A72C-E9D1-5790-A2CF-D98C79FD46EC

https://zoobank.org/83E65E19-56DE-4A30-999F-0252BE24E7F7

Figs 3 , 4 , 5

Orbiniella petersenae : Parapar et al. 2015: 333–343, figs 3–9 (in part).

##### Clade.

Shallow.

##### Type material examined.

***Holotype***ZMBN 157444 (DNA voucher Orbi43). ***Paratypes***ZMBN 157397 (1 paratype, DNA voucher Orbi32); ZMBN 157398 (1 paratype, DNA voucher Orbi33); ZMBN 157399 (1 paratype, DNA voucher Orbi30); ZMBN 157400 (1 paratype, DNA voucher Orbi31); ZMBN 157401 (3 paratypes); ZMBN 157402 (22 paratypes); ZMBN 157403 (1 paratype, DNA voucher Orbi28); ZMBN 157404 (1 paratype, DNA voucher Orbi27); ZMBN 157434 (1 paratype); ZMBN 157435 (1 paratype); ZMBN 157436 (1 paratype on SEM stub); ZMBN 157437 (1 paratype); ZMBN 157438 (1 paratype); ZMBN 157439 (1 paratype); ZMBN 157440 (1 paratype on SEM stub); ZMBN 157441 (1 paratype); ZMBN 157442 (5 paratypes); ZMBN 157443 (1 paratype); ZMBN 157445 (1 paratype, DNA voucher LC-57); ZMBN 157446 (1 paratype); ZMBN 157646 (1 paratype); SMF 32601 (1 paratype, DNA voucher Orbi4); SMF 32637 (1 paratype); SMF 32639 (1 paratype, DNA voucher Orbi17); SMF 32665 (1 paratype, DNA voucher Orbi5).

##### Other material examined.

ZMBN 95697 (1 spm); ZMBN 95728 (E-voucher POLNB1250-14); ZMBN 95735 (E-voucher POLNB1257-14); ZMBN 157668 (E-voucher ICBA263-16); ZMBN 157669 (E-voucher ICBA264-16); ZMBN 157670 (E-voucher NBAAV696-23); ZMBN 157671 (E-voucher NBAAV686-23); ZMBN 157672 (E-voucher NBAAV697-23); ZMBN 157673 (E-voucher NBAAV692-23); ZMBN 157674 (E-voucher NBAAV693-23); 18 specimens from the *O.petersenae* sensu lato type series: IINH 34892 (2 spms); IINH 34894 (16 spms).

##### Diagnosis.

An *Orbiniella* with segmental annulation pattern as follows: one narrow annulus between parapodium 1 and 6, two narrow annuli between parapodia from parapodium 6 until 8, and three narrow annuli between parapodia from parapodium 8 until pygidium. Acicular spines short and stout, up to two in both noto- and neuropodia. Pygidium with four long anal lobes assembled together.

##### Type locality.

**Figure 3.**
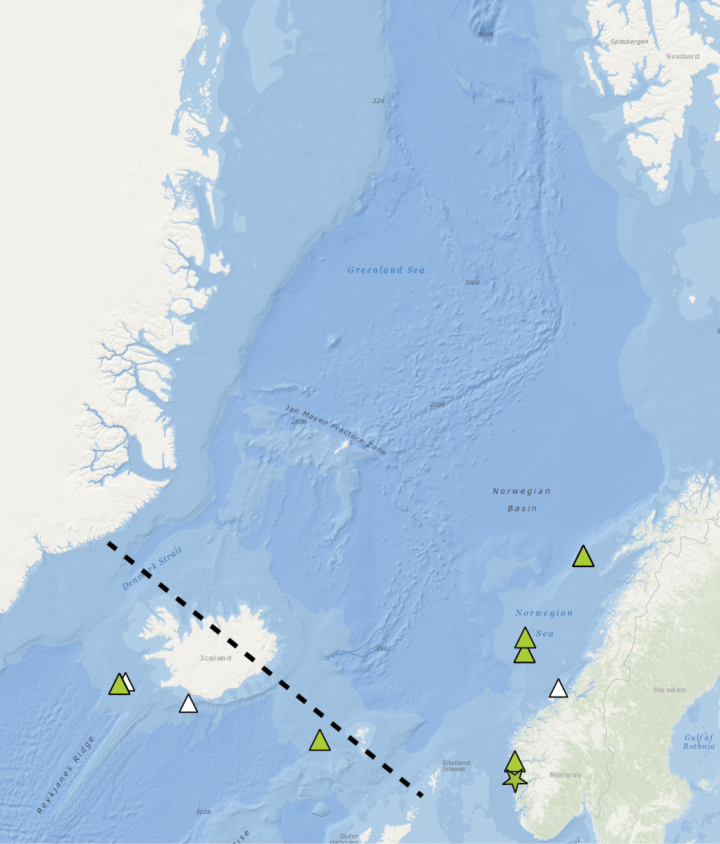
Distribution of *Orbiniellagriegi* Meca & Budaeva, sp. nov. Black lines – Greenland-Scotland Ridge (GSR), star – type locality, coloured triangles – stations with examined morphology and molecular data, white triangles – stations with examined morphology.

Basvika, Bergen area, Norwegian West coast, 60.3959, 5.1492, 172 m (Fig. 3).

##### Description

(based on type specimens). Holotype complete with 32 chaetigers, 4.9 mm long and 0.4 mm wide at level of chaetiger 7. Body elongated and narrow, uniformly wide, slightly narrowing in pre-pygidial area. Pigmentation lacking in all analysed specimens.

**Figure 4.**
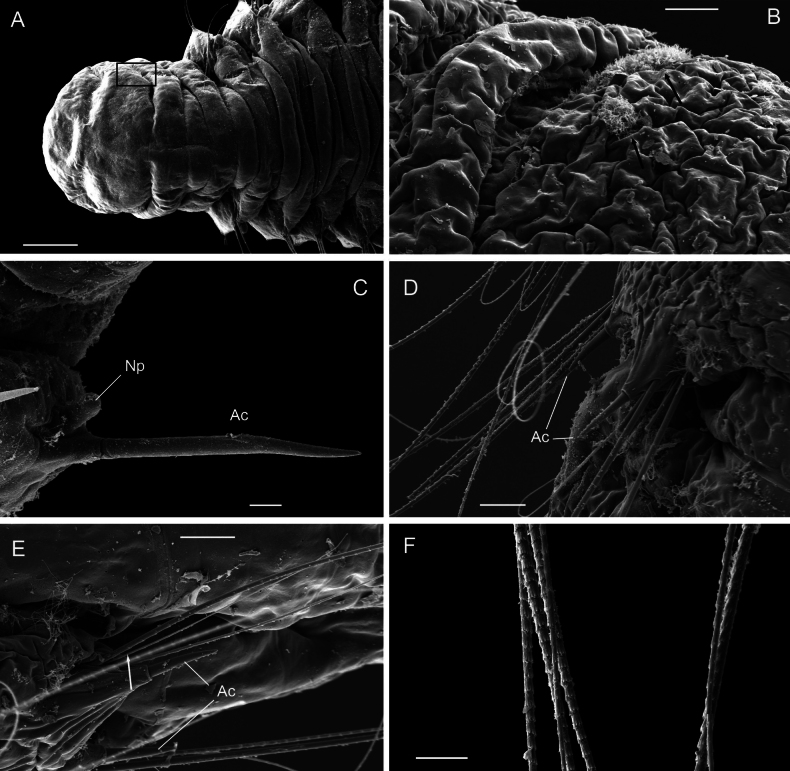
*Orbiniellagriegi* Meca & Budaeva, sp. nov., SEM**A** paratype ZMBN 157440, anterior end, dorsal view **B** paratype ZMBN 157440, detail of right lateral side of prostomium showing presumable nuchal organ (insert of **A**) **C** paratype ZMBN 157440, notopodium of chaetiger 20 showing notopodial lobe and acicular spine **D** paratype ZMBN 157436, parapodium of chaetiger 7 showing acicular spines disposed in vis-à-vis position and capillaries **E** paratype ZMBN 157436, neuropodium of chaetiger 9 showing acicular spines and capillaries **F** paratype ZMBN 157440, capillary chaetae. Black arrows in **B**: Lateral ciliary spots; White arrow in **E**: Start of crenulation in capillary chaeta. Abbreviations: Ac: acicular spines; Np: notopodial lobe. Scale bars: 100 μm (**A**); 20 μm (**B, D, E**); 10 μm (**C, F**).

**Figure 5.**
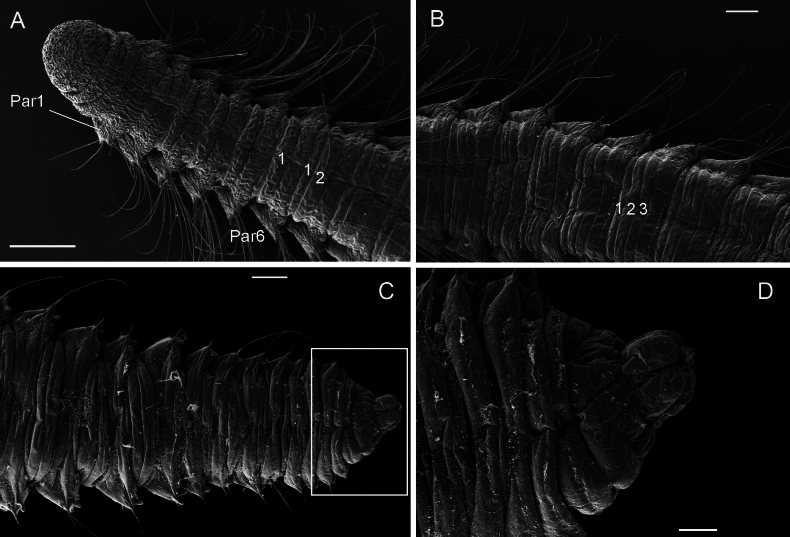
*Orbiniellagriegi* Meca & Budaeva, sp. nov., SEM**A** paratype ZMBN 157436, anterior end, dorsal view showing narrow annuli between parapodia 1 and 8 **B** paratype ZMBN 157436, mid body, dorsal view showing narrow annuli between parapodia 9 and 16 **C** paratype ZMBN 157440, posterior end, dorsal view **D** paratype ZMBN 157440, detail of pygidium (insert of **C**). Numbers show the segmental annulation pattern. Abbreviations: Par1: first parapodium; Par6: sixth parapodium. Scale bars: 200 μm (**A**); 100 μm (**B–D**).

Prostomium broad with rounded anterior margin, without eyespots (Fig. 4A). SEM micrographs showed two lateral, inconspicuous ciliary spots on both sides of prostomium, presumably nuchal organs (Fig. 4B). Peristomium with two prominent achaetous segments, second shorter than first, distinctly separated from each other and from first chaetiger by narrow annulus (Fig. 4A). From chaetiger 7–10 onwards segments becoming longer, more square-shaped. Segmental annulation in following pattern: one narrow annulus between parapodium 1 and 6, two narrow annuli between parapodia from parapodium 6 until 8, and three narrow annuli between parapodia from parapodium 8 until pygidium (Fig. 5A–C). Segmental annulation less defined in pre-pygidial region.

Parapodia biramous, triangular-like, of similar size throughout body. Postchaetal neuropodial lobe absent. Digitate postchaetal notopodial lobes short and thick, from chaetiger 1 (Fig. 4C). Crenulated capillary chaetae and spines present in both rami from CH1. Capillaries equal in length to body width and numerous (6–8 per bundle) in anterior segments; shorter and reduced in number in posterior segments. Capillary chaetae with crenulation occurring on one side along whole chaeta or along half of its length (Fig. 4E). Spines short, stout, smooth, one or two per ramus (Fig. 4D). Last eight or nine chaetigers short and few achaetous (Fig. 5C). Pygidium with four long anal lobes assembled together (Fig. 5D).

##### Variation.

The holotype and most of the paratypes shared the same morphology. However, some paratypes collected from the Norwegian shelf presented more numerous (7–10 per bundle) and longer (longer than body width) capillaries in anterior segments compared to specimens from the type locality

##### Remarks.

*Orbiniellagriegi* sp. nov. is morphologically nearly identical to the other three species described in this work: *O.mayhemi* sp. nov., *O.parapari* sp. nov. and *O.petersenae* sensu stricto, which led to combining them into a single species by Parapar et al. (2015). However, the four species display differences in the segmental annulation pattern in the anterior body (i.e., an anterior-most region with one narrow annulus between parapodia followed by a short region with two narrow annuli between parapodia in *O.griegi* sp. nov., an anterior-most region with one narrow annulus between parapodia followed by a region bearing two narrow annuli between parapodia that extends to mid-posterior body in *O.mayhemi* sp. nov., an anterior-most region with one narrow annulus between parapodia followed by a region bearing two narrow annuli between parapodia that extends to mid-anterior body in *O.parapari* sp. nov., and an anterior-most region bearing one and two narrow annuli between parapodia followed by a region with three narrow annuli between parapodia in *O.petersenae* sensu stricto) (Fig. 2). *Orbiniellagriegi* sp. nov. also differs from the other species by having one or two short and stout acicular spines while *O.mayhemi* sp. nov. has 1–3 short spines, *O.parapari* sp. nov. has 1–6 long and thin spines, and *O.petersenae* sensu stricto bears 1–5 short spines. It can further be distinguished by having long anal lobes assembled together while *O.parapari* sp. nov. and *O.petersenae* sensu stricto bear short lobes on their pygidia (Fig. 2). The shape of the lobes is unknown in *O.mayhemi* sp. nov. (see also Table 1 with comparison of the Nordic *Orbiniella* species). *Orbiniellagriegi* sp. nov. also showed no intraspecific variation in the shape of prostomium, peristomium and notopodia. *Orbiniellagriegi* sp. nov. resembles *O.mayhemi* sp. nov. in having a broad prostomium, but differs in the second peristomial segment being shorter than the first instead of the first segment being shorter than the second. Furthermore, both *O.griegi* sp. nov. and *O.mayhemi* sp. nov. bear digitate notopodial lobes, but showing slightly narrowing basal part in the latter.

**Table 1.** Discriminatory characters of the NE Atlantic *Orbiniella*. Abbreviations: Af, Available fragment; CH, Chaetiger; NA, Narrow annuli between parapodia; Par, Parapodium; Pg, Pygidium.

Character	*Orbiniellagriegi* sp. nov.	*Orbiniellamayhemi* sp. nov.	*Orbiniellaparapari* sp. nov.	*Orbiniellapetersenaesensu stricto* Parapar, Moreira & Helgason, 2015
Prostomium	Broad	Broad	Broad or elongate	Broad or elongate
Nuchal organs	In patches	Not observed	One single congregation	One single congregation
Peristomial rings	First wider than second	first narrower than second	Both with same length or first narrower than second	Both with same length or first narrower than second
Segmental annulation pattern	One NA from Par 1 until 6, two NA from Par 6 until 8, and three NA from Par 8 until Pg	One NA from Par 1 until 5-6 and two NA from Par 5-6 until end of Af of the most complete specimen	One NA from Par 1 until 5-6, two NA from Par 5-6 until 10-14, and three NA from Par 10-14 until Pg	One NA between Par 1 and 2, two NA from Par 2 until 5-6, and three NA from Par 5-6 until Pg
Shape parapodia	Triangular	Rounded in the first chaetigers and triangular from CH7–CH10	Triangular or rounded in the first chaetigers and triangular from CH7–CH10	Triangular or rounded in the first chaetigers and triangular from CH7–CH10
Shape notopodia	Digitate (short)	Digitate (short) with slightly narrowing basal part	Digitate (short or long)	Digitate (short or long)
Capillaries	6-10 per bundle	6-10 per bundle	7-10 per bundle	7-10 per bundle
Spines	1-2 per ramus (short)	1-3 per ramus (short)	1-6 per ramus (long)	1-5 per ramus (short)
Pygidium	Four long lobes assembled together	Not observed	Four short and thick lobes	Four short lobes
Distribution	Norwegian shelf, Faroe-Iceland Ridge and SW Iceland	SW and SE Iceland	Iceland Sea and Norwegian Sea	Iceland Sea, Norwegian Sea and southern Greenland Sea
Depth	171-781 m	913-2505 m	1811-2832 m	1053-2407 m

*Orbiniellagriegi* sp. nov., together with *O.marionensis* Gillet, 1999, are unique among the known shallow water *Orbiniella* species in having notopodial postchaetal lobes and in having acicular spines in both noto- and neuropodia, as in the seven deep-sea species: *O.andeepia* Narayanaswamy & Blake, 2005, from Antarctica, *O.longilobata* Blake, 2020, from South China Sea, *O.rugosa* Blake, 2020, from South China Sea, *O.tumida* Blake, 2020, from the California continental slope, *O.abyssalis* Blake, 2020, from the abyssal Pacific Ocean, *O.armata* Blake, 2021, from off South Carolina, and *O.mimica* Blake, 2021, from NW Atlantic. *Orbiniellagriegi* sp. nov. differs from these species in having three narrow annuli between parapodia and a pygidium with four anal lobes assembled together and from *O.abyssalis*, additionally, in having two peristomial segments instead of a single peristomial segment.

##### Distribution.

Norwegian coastal areas and shelf, Faroe-Island Ridge, and SW Iceland, 171–781 m (Fig. 3).

##### Etymology.

This species is named in honour of Edvard Grieg, the Norwegian musician born and raised in Bergen, the city where the present study was conducted.

#### ﻿ Orbiniella mayhemi

Taxon classificationAnimaliaOrbiniidaOrbiniidae

Meca & Budaeva, sp. nov.

6BE73540-FD8B-531E-8382-42CEB978411A

https://zoobank.org/E68E6555-FA57-436A-BA90-C6EF1A4C2C32

Figs 6 , 7

##### Clade.

Deep 3.

##### Type material examined.

***Holotype***SMF 32627 (DNA voucher Orbi19). ***Paratypes***SMF 32588 (1 paratype); SMF 32628 (1 paratype on SEM stub); SMF 32630 (1 paratype); SMF 32640 (1 paratype on SEM stub, DNA voucher Orbi18); ZMBN 157433 (1 paratype, DNA voucher Orbi3).

##### Other material examined.

ZMBN 157432 (3 spms).

##### Diagnosis.

An *Orbiniella* with segmental annulation pattern as follows: one narrow annulus between parapodia from parapodium 1 until 5 or 6 and two narrow annuli between parapodia from parapodium 5 or 6 until end of available fragment of the most complete specimen. Acicular spines short and stout, up to three in both noto- and neuropodia. Pygidium not observed.

##### Type locality.

**Figure 6.**
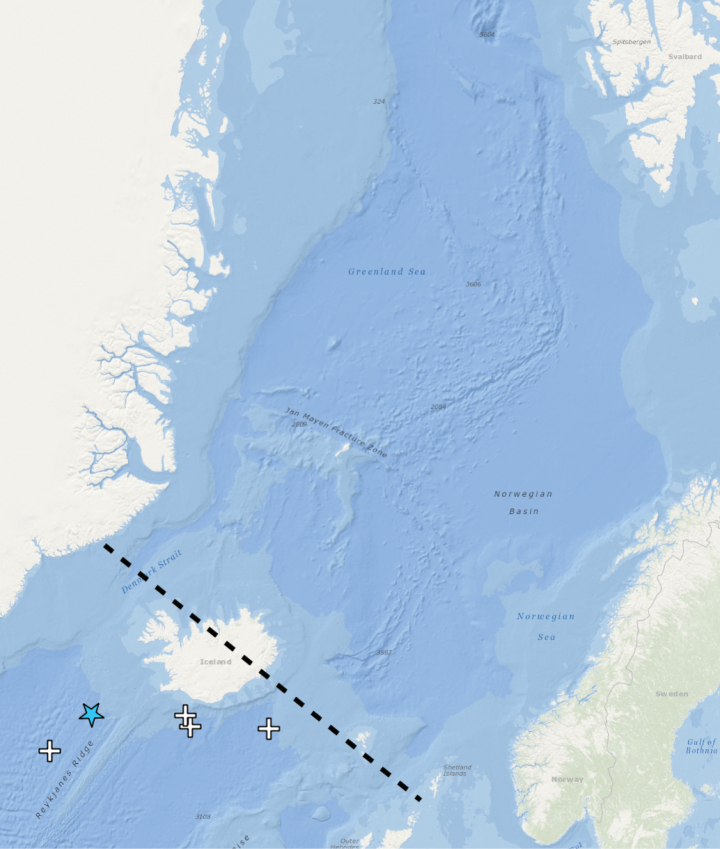
Distribution of *Orbiniellamayhemi* Meca & Budaeva, sp. nov. Black lines – Greenland-Scotland Ridge (GSR), star – type locality and the station with examined morphology and molecular data, white plusses – stations with examined morphology.

Irminger Basin, SW Iceland, NE Atlantic, 62.9888, -28.0950, 1588 m (Fig. 6).

##### Description

(based on type specimens). Holotype incomplete with 14 chaetigers, 3.0 mm long and 0.36 mm wide at level of chaetiger 6. Body elongated and narrow, uniformly wide. Pigmentation lacking in all analysed specimens.

**Figure 7.**
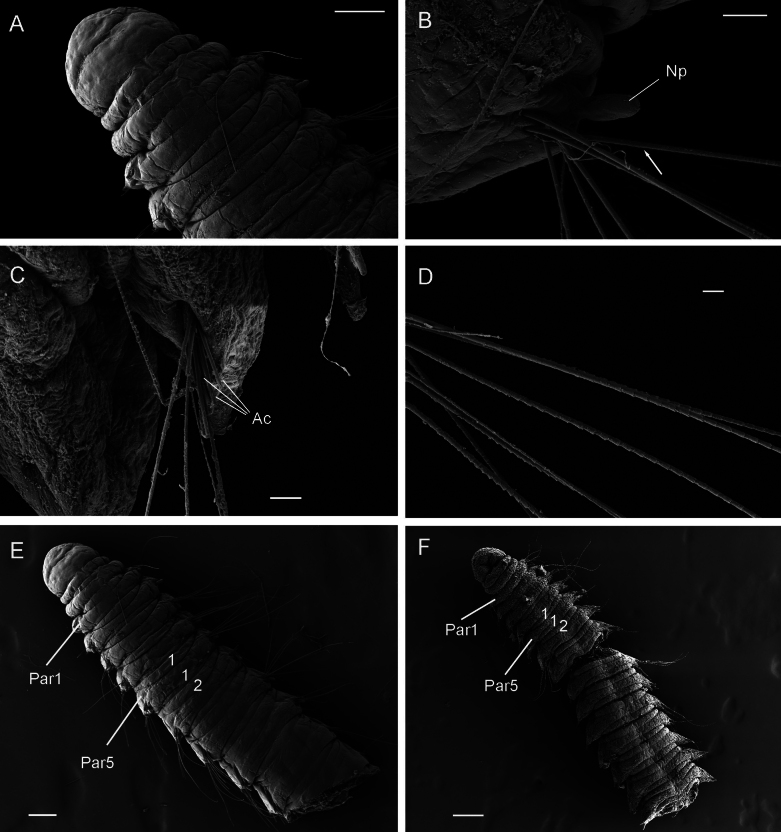
*Orbiniellamayhemi* Meca & Budaeva, sp. nov. SEM**A** paratype SMF 32640, anterior end, dorsal view **B** paratype SMF 32640, notopodium of chaetiger 7 showing notopodial lobe and capillaries **C** paratype SMF 32628, neuropodium of chaetiger 5 showing acicular spines and capillaries **D** paratype SMF 32640, capillary chaetae **E** paratype SMF 32640, anterior fragment showing narrow annuli between parapodia 1 and 10, dorsal view **F** paratype SMF 32628, anterior fragment showing narrow annuli between parapodia 1 and 14, ventral view. White arrow in **B**: Start of crenulation in capillary chaeta. Abbreviations: Ac: acicular spines; Np: notopodial lobe; Par1: first parapodium; Par5: fifth parapodium. Scale bars: 100 μm (**A, E**); 20 μm (**B, C**); 10 μm (**D**); 200 μm (**F**).

Prostomium broad with rounded anterior margin, without eyespots or nuchal organs (Fig. 7A). Peristomium with two prominent achaetous segments, first segment shorter than second segment, distinctly separated from each other and first chaetiger by a narrow annulus (Fig. 7A). Anterior chaetigers short, becoming longer and more square-shaped from chaetiger 7–10 onwards. Segmental annulation pattern: one narrow annulus between parapodia from parapodium 1 until 5 or 6 and two narrow annuli between parapodia from parapodium 5 or 6 until end of available fragment of most complete specimen (Fig. 7E, F). Posterior part and pygidium not observed.

Parapodia biramous, wider than long, with postchaetal notopodial lobes short, digitate with slightly narrowing basal part, from chaetiger 1 (Fig. 7B). Postchaetal neuropodial lobes absent. Crenulated capillary chaetae and acicular spines present in both rami from chaetiger 1. Capillaries equal in length to body width and numerous (6–8 per bundle) in anterior segments; shorter and reduced in number in posterior segments. Capillary chaetae with crenulation occurring on one side along whole chaeta or along half of its length (Fig. 7D). Acicular spines short, stout, and smooth, up to three per ramus (Fig. 7C).

##### Variation.

The holotype and most of the paratypes shared the same morphology. However, some paratypes presented more numerous (7–10 per bundle) and longer (longer than body width) capillaries in anterior segments.

##### Remarks.

*Orbiniellamayhemi* sp. nov. differs from the other three species described in this work (i.e., *O.griegi* sp. nov., *O.parapari* sp. nov., and *O.petersenae* sensu stricto) in having a body region bearing two narrow annuli between parapodia more extensive than in the other three species. *Orbiniellamayhemi* sp. nov. also bears short, stout, and smooth spines as *O.griegi* sp. nov. and *O.petersenae* sensu stricto, but differing in number (i.e., 1–3 per ramus in *O.mayhemi* sp. nov.; 1 or 2 in *O.griegi* sp. nov., and 1–5 in *O.petersenae* sensu stricto).

Similarly to *O.griegi* sp. nov., *Orbiniellamayhemi* sp. nov. resembles the seven deep-sea congeners *O.andeepia*, *O.longilobata*, *O.rugosa*, *O.tumida*, *O.abyssalis*, *O.armata*, and *O.mimica* in having notopodial postchaetal lobe and in having acicular spines in both noto- and neuropodia. However, *O.mayhemi* sp. nov. differs from *O.abyssalis* in having two peristomial segments instead of a single peristomial segment. Among these seven deep-sea species, *O.mimica* is the closest geographically with *O.mayhemi* sp. nov. (NW vs NE Atlantic, respectively), being similar in having one or two narrow annuli between parapodia and up to three acicular spines. *Orbiniellamimica* differs, however, in presenting a papillated dorsal surface on the prostomium and numerous glands in the parapodia and chaetal segments instead of a smooth surface and a uniform digitate notopodial postchaetal lobe instead of a lobe narrowing basally.

##### Distribution.

From SW to SE Iceland, 913–2505 m (Fig. 6).

##### Etymology.

The species is named in honour to the Norwegian Black Metal band from Oslo, Mayhem, one of the bands that most contributed to the development of the Norwegian Black Metal in the 90-s. MAM was listening to their music to endure the darkest hours in the lab.

#### ﻿ Orbiniella parapari

Taxon classificationAnimaliaOrbiniidaOrbiniidae

Meca & Budaeva, sp. nov.

7D61251D-A090-562E-9F19-351C8F029416

https://zoobank.org/CD7DFB4F-57FD-46C4-A575-1135877C7B91

Figs 8 , 9 , 10

Orbiniella petersenae : Parapar et al. (2015): 333–343, figs 3–9 (in part).

##### Clade.

Deep 2.

##### Type material examined.

***Holotype***ZMBN 157405 (DNA voucher Orbi40). ***Paratypes***ZMBN 157406 (8 paratypes); ZMBN 157407 (1 paratype on SEM stub); ZMBN 157408 (9 paratypes); ZMBN 157409 (2 paratypes on SEM stub); ZMBN 157410 (1 paratype on SEM stub); ZMBN 157411 (1 paratype, DNA voucher Orbi37); ZMBN 157412 (1 paratype on SEM stub, DNA voucher Orbi38); ZMBN 157413 (1 paratype on SEM stub, DNA voucher Orbi38); ZMBN 157414 (3 paratypes); ZMBN 157415 (4 paratypes); ZMBN 157416 (1 paratype); ZMBN 157417 (1 paratype); ZMBN 157418 (2 paratypes); ZMBN 157424 (3 paratypes); ZMBN 157425 (8 paratypes); ZMBN 157426 (2 paratypes); ZMBN 157429 (2 paratypes on SEM stub); SMF 32659 (3 paratypes); SMF 32662 (5 paratypes); SMF 32644 (1 paratype); SMF 32653 (4 paratypes); SMF 32655 (5 paratypes); SMF 32664 (1 paratype, DNA voucher Orbi26); SMF 32666 (4 paratypes); SMF 32667 (1 paratype, DNA voucher Orbi10); SMF 32668 (1 paratype on SEM stub, DNA voucher Orbi11); SMF 32669 (1 paratype on SEM stub, DNA voucher Orbi12); SMF 32680 (1 paratype).

##### Other material examined.

ZMBN 157427 (4 spms); ZMBN 157428 (45 spms); ZMBN 157430 (30 spms); ZMBN 157431 (4 spms); ZMBN 157675 (E-voucher NBAAV687-23); ZMBN 157676 (E-voucher NBAAV689-23); ZMBN 157677 (E-voucher NBAAV691-23); ZMBN 157678 (E-voucher NBAAV690-23); ZMBN 157679 (E-voucher NBAAV694-23); ZMBN 157680 (E-voucher NBAAV688-23); SMF 32652 (8 spms); SMF 32654 (2 spms); SMF 32657 (39 spms); IceAGE sample DZMB-HH 33669 (E-voucher NBAAV668-23); IceAGE sample DZMB-HH 49578 (E-voucher NBAAV671-23); IceAGE sample DZMB-HH 49614 (E-voucher NBAAV669-23); IceAGE sample DZMB-HH 49614 (E-voucher NBAAV670-23); 56 specimens from the *O.petersenae* sensu lato type series: IINH 43199 (40 spms), IINH 43197 (16 spms).

##### Diagnosis.

An *Orbiniella* with segmental annulation pattern as follows: one narrow annulus between parapodia from parapodium 1 until 5 or 6, two narrow annuli between parapodia from parapodium 5 or 6 until 10–14, and three narrow annuli between parapodia from parapodium 10–14 until pygidium. Acicular spines long and thin, up to six in both noto- and neuropodia. Pygidium with four short and thick anal lobes.

##### Type locality.

**Figure 8.**
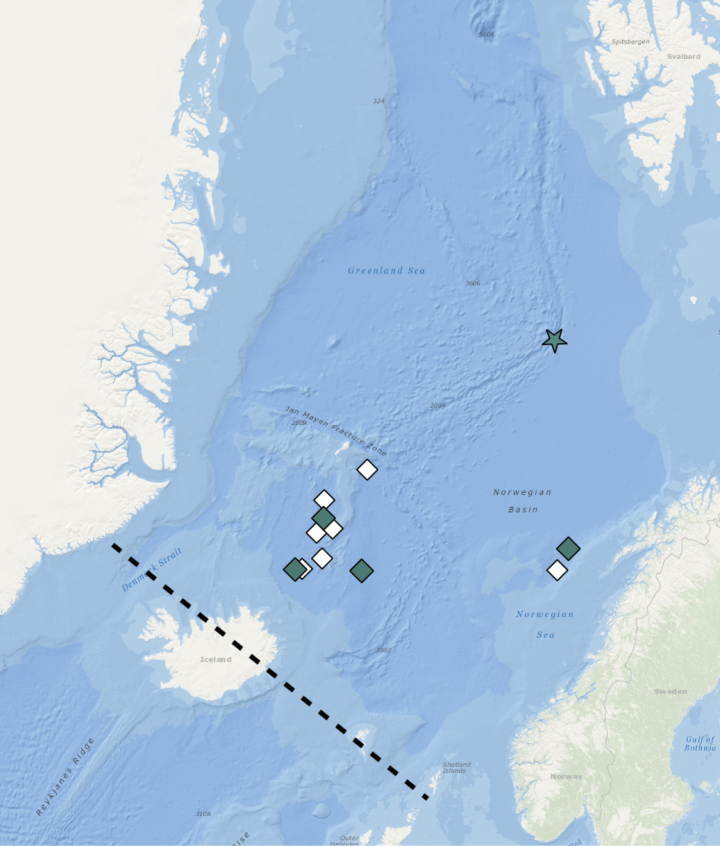
Distribution of *Orbiniellaparapari* Meca & Budaeva, sp. nov. Black lines – Greenland-Scotland Ridge (GSR), star – type locality, coloured rhombuses – stations with examined morphology and molecular data, white rhombuses – stations with examined morphology.

Loki’s Castle, Arctic Mid-Ocean Ridge, 73.5663, 8.1610, 2450 m (Fig. 8).

##### Description

**Figure 9.**
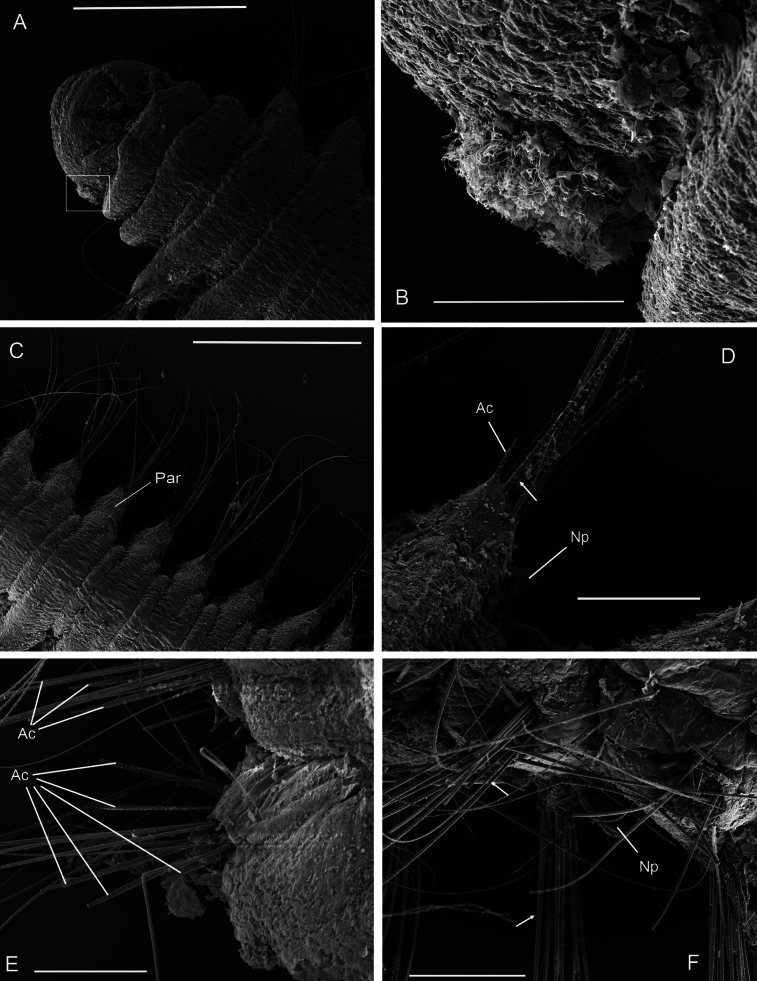
*Orbiniellaparapari* Meca & Budaeva, sp. nov., SEM**A** paratype ZMBN 157429, anterior end, ventral view **B** paratype ZMBN 157429, detail of right lateral side of prostomium showing presumed nuchal organ (insert of A) **C** paratype ZMBN 157429, first eight anterior parapodia **D** paratype ZMBN 157429, neuropodium of chaetiger 3 showing acicular spines, capillaries, and notopodial lobe from dorsal ramus **E** paratype ZMBN 157410, neuropodium of chaetiger 17 showing acicular spines **F** paratype ZMBN 157412, notopodium of chaetiger 7 showing notopodial lobe and capillaries. White arrows in **D** and **F**: Start of crenulation in capillary chaeta. Abbreviations: Ac: acicular spines; Np: notopodial lobe; Par: parapodia. Scale bars: 200 μm (**A**); 30 μm (**B**); 300 μm (**C**); 50 μm (**D**); 100 μm (**E, F**).

**Figure 10.**
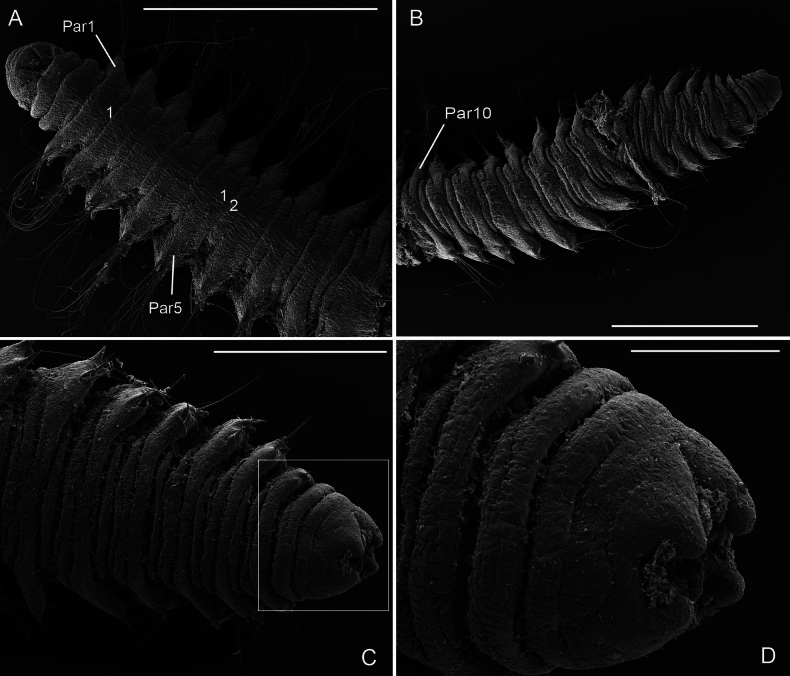
*Orbiniellaparapari* Meca & Budaeva, sp. nov., SEM of paratypes ZMBN 157429 **A** anterior end showing narrow annuli between parapodia 1 and 9, ventral view **B** mid body and posterior end, ventral view **C** posterior end, ventral view **D** detail of pygidium (insert of C). Numbers show the segmental annulation pattern. Abbreviations: Par1: first parapodium; Par5: fifth parapodium; Par10: tenth parapodium. Scale bars: 500 μm (**A, B**); 300 μm (**C**); 100 μm (**D**).

(based on type specimens). Holotype complete with 29 chaetigers, 4.3 mm long and 0.5 mm wide at level of chaetiger 6. Body short and thick, uniformly wide, narrowing in preanal area. Pigmentation lacking. Prostomium broad with rounded anterior margin, without eyespots (Fig. 9A). Peristomium with two prominent achaetous segments, first peristomial segment shorter than second, distinctly separated from each other and first chaetiger by narrow annulus (Fig. 9A). No conspicuous nuchal organs observed. Anterior segments short, becoming longer, more square-shaped from chaetiger 8 onwards. Segmental annulation of following pattern: one narrow annulus between parapodia from parapodium 1 until 5 or 6, two narrow annuli between parapodia from parapodium 5 or 6 until 10–14, and three narrow annuli between parapodia from parapodium 10–14 until pygidium (Fig. 10A, B). Segmental annulation less defined in pre-pygidial region.

Parapodia biramous, wider than long (Fig. 9C), with postchaetal notopodial lobe digitate, short, and thick from chaetiger 1. Postchaetal neuropodial lobe absent. Crenulated capillary chaetae and acicular spines present in both rami from chaetiger 1. Capillaries in anterior segments longer than body width and numerous (7–10 per bundle), in posterior segments shorter and reduced in number. Acicular spines long, thin, and smooth, up to six per ramus (Fig. 9E).

Last five or six posterior segments slightly shorter and last two achaetous (Fig. 10C). Pygidium with four short and thick anal lobes (Fig. 10D).

##### Variation.

The holotype and all paratypes collected from the Loki’s Castle vent field shared the same morphology. Other paratypes collected from the Iceland Sea and the Norwegian Sea showed variation in the shape of prostomium, peristomium and notopodia.

Some of the specimens collected in the IceAGE project displayed three different patterns of dorsal segmental pigmentation: lateral patches from chaetiger 3 to the rest of the body; transversal bands through all the segment in chaetigers 3, 4, and 5 and in patches in the remaining chaetigers; bands in chaetiger 3, 4, and 5 without pigmentation in the rest of the body. The remaining pigmentation might be due to more recent collection date of the IceAGE specimens comparable to the type material.

The shape of prostomium varied between being broad (Figs 9A, 10A) or elongate, with a rounded anterior margin. Poorly defined brownish eyespots were observed in some of the specimens from the *O.petersenae* sensu lato type series material. Most of the paratypes had the first segment of the peristomium shorter than the second, as in the holotype, but a few paratypes were with both segments equal in length. Notopodia varied between being digitate, long, and thin or digitate, short, and thick (Fig. 9D, F; Table 1).

SEM micrographs of several paratypes showed one single prominent lateral ciliary congregation at each side of the prostomium, which we interpreted as nuchal organs (Fig. 9B). Capillary chaetae with crenulation occurring on one side along the whole chaeta (Fig. 9D) or along half of its length (Fig. 9F). Crenulation can be clear along the whole chaeta or become more obvious distally. The specimens from the *O.petersenae* sensu lato type series and the holotype showed capillaries longer than body width in anterior segments as Parapar et al. (2015) stated. However, other specimens from the material collected in this study presented shorter capillaries equal to body width.

##### Remarks.

Among the Nordic *Orbiniella* species described here, *O.parapari* sp. nov. closely resembles *O.petersenae* sensu stricto, with which it has overlapping geographical ranges (i.e., Iceland Sea and Norwegian Sea). Both species are similar in bearing high number of acicular spines (i.e., 1–5 per ramus in the former and 1–6 in the latter), which, however, appear longer and thinner in *O.parapari* sp. nov. Also, both species share a pygidium with four short, thick anal lobes, although the lobes are slightly shorter and thicker in *O.parapari* sp. nov. Both species also have one, two, and three narrow annuli between parapodia; however, their pattern of progression along the body differs: an anterior-most region with one narrow annulus between parapodia followed by a region bearing two narrow annuli between parapodia that extends to mid-anterior body, and continuing with three narrow annuli between parapodia in *O.parapari* sp. nov.; and an anterior-most region bearing one and two narrow annuli between parapodia followed by an extensive region with three narrow annuli between parapodia in *O.petersenae* sensu stricto. *Orbiniellagriegi* sp. nov. also shows one to three narrow annuli between parapodia (with a different pattern, see the description of *O.griegi*), but has fewer (1 or 2) acicular spines and a pygidium with four long anal lobes assembled together.

As in the case of the other Nordic *Orbiniella* species described here, *O.parapari* sp. nov. shares a number of morphological characters with the seven deep-sea congeners: *O.andeepia*, *O.longilobata*, *O.rugosa*, *O.tumida*, *O.abyssalis*, *O.armata*, and *O.mimica* (see details on similarities and dissimilarities of these species with respect to *O.mayhemi* sp. nov.). However, *O.parapari* sp. nov. differs from these seven species in having three intersegmental rings, and with the exception of *O.mimica*, a pygidium bearing four short anal lobes. *Orbiniellamimica* also bears four lobes in the pygidium, but they are much shorter than in *O.parapari* sp. nov. and each of them is accompanied by a long, thin anal cirrus. Moreover, *O.mimica* presents a unique papillated dorsal surface and a smaller number (up to 3) of acicular spines than in *O.parapari* sp. nov. (up to 6 spines).

##### Distribution.

Iceland Sea and Norwegian Sea, 1811–2832 m (Fig. 8).

##### Etymology.

This species is dedicated to the Spanish polychaetologist Dr. Julio Parapar, who described the first *Orbiniella* species in the North Atlantic waters, *Orbiniellapetersenae* Parapar, Moreira & Helgason, 2015.

#### ﻿ Orbiniella petersenae

Taxon classificationAnimaliaOrbiniidaOrbiniidae

Parapar, Moreira & Helgason, 2015, sensu stricto

5727AA57-846F-57CB-A3A5-1FF6AFB3B0AB

Figs 11 , 12 , 13

Orbiniella petersenae : Parapar et al. 2015: 333–343, figs 3–9 (in part).

##### Clade.

Deep 1.

##### Type material examined.

***Holotype***IINH 35670. ***Paratypes***IINH 29822 (27 paratypes), IINH 34897 (1 posterior end of a paratype on SEM stub), IINH 34899 (9 paratypes), IINH 35671 (36 paratypes), IINH 35672 (1 posterior end of a paratype), IINH 35673 (3 paratypes), IINH 35699 (6 paratypes on SEM stub).

##### Other material examined.

ZMBN 157647 (35 spms); ZMBN 157648 (1 spms); ZMBN 157649 (1 spm on SEM stub); ZMBN 157650 (2 spms on SEM stub); ZMBN 157651 (1 spm); ZMBN 157652 (60 spms); ZMBN 157653 (3 spms); ZMBN 157654 (12 spms); ZMBN 157655 (10 spms); ZMBN 157656 (1 spm); ZMBN 157657 (28 spms); ZMBN 157658 (1 spm); ZMBN 157659 (11 spms); ZMBN 157660 (7 spms); ZMBN 157661 (38 spms); ZMBN 157662 (322 spms); ZMBN 157663 (2 spms on SEM stub); SMF 32584 (2 spms); SMF 32589 (4 spms); SMF 32629 (6 spms); SMF 32632 (23 spms); SMF 32633 (4 spms); SMF 32634 (3 spms); SMF 32635 (3 spms); SMF 32636 (52 spms); SMF 32646 (17 spms); SMF 32647 (41 spms); SMF 32648 (2 spms); SMF 32670 (DNA voucher Orbi2 on SEM stub); SMF 32671 (DNA voucher Orbi7); SMF 32672 (DNA voucher Orbi8); SMF 32673 (DNA voucher Orbi9); SMF 32675 (DNA voucher Orbi1); SMF 32676 (DNA voucher Orbi13); SMF 32677 (DNA voucher Orbi14); SMF 32678 (DNA voucher Orbi15 on SEM stub); SMF 32679 (DNA voucher Orbi16 on SEM stub); SMF 32660 (DNA voucher Orbi20); IceAGE sample DZMB-HH 33669 (E-voucher NBAAV667-23).

##### Diagnosis.

An *Orbiniella* with segmental annulation pattern as follows: one narrow annulus between parapodium 1 and 2, two narrow annuli between parapodia from parapodium 2 until 5 or 6, and three narrow annuli between parapodia from parapodium 5 or 6 until pygidium. Acicular spines short and stout, up to five in both noto- and neuropodia. Pygidium with four short anal lobes.

##### Type locality.

**Figure 11.**
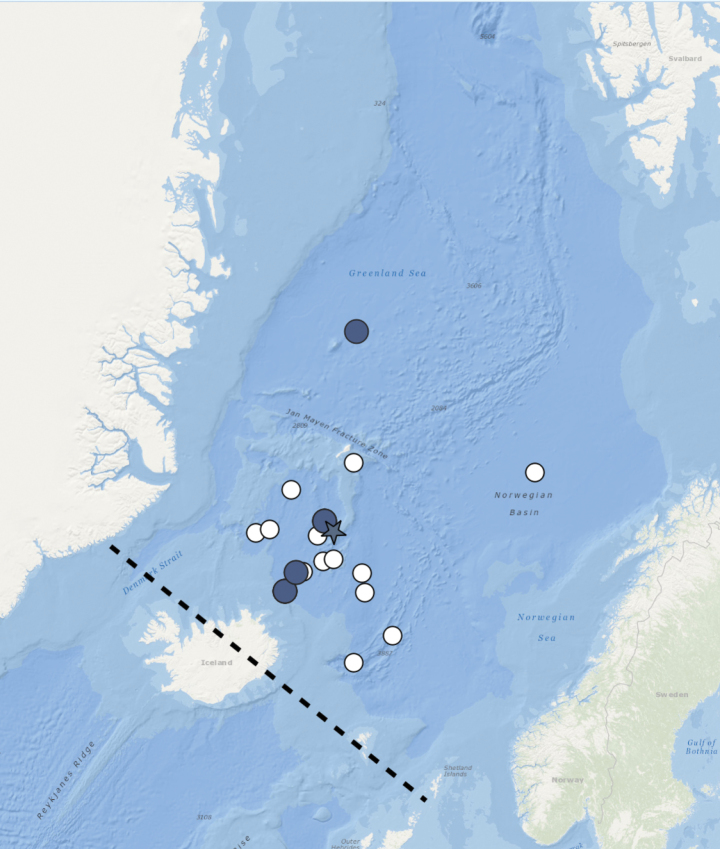
Distribution of *Orbiniellapetersenae* sensu stricto. Black lines – Greenland-Scotland Ridge (GSR), star – type locality, coloured circles – stations with examined morphology and molecular data, white circles – stations with examined morphology.

Jan Mayen microcontinent, Iceland Sea, 68.8285, -9.2403, 1849 m (Fig. 11).

##### Remarks.

The morphology of the holotype fully agrees with the original description. The analysis of the IINH paratypes and the new material in this study under a light microscope and SEM allowed elucidating the degree of variability in some morphological characters not discussed by Parapar et al. (2015).

**Figure 12.**
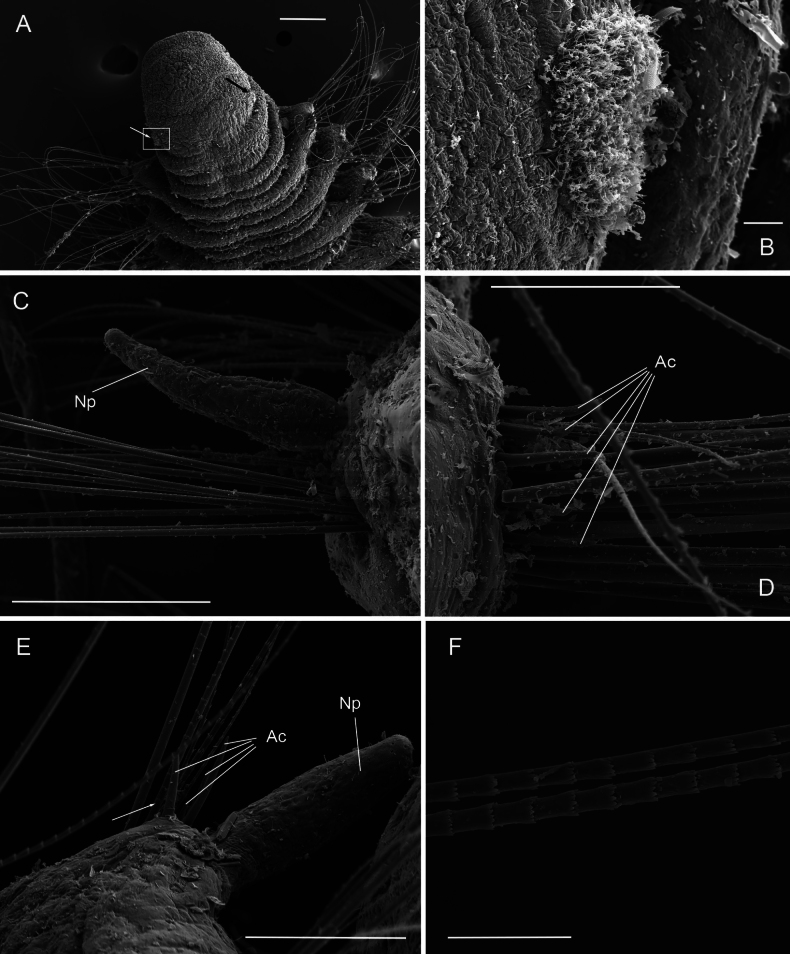
*Orbiniellapetersenae* sensu stricto, SEM**A** paratype ZMBN 157649, anterior end, dorsal view **B** paratype ZMBN 157649, detail of left lateral side of prostomium showing presumed nuchal organs (insert of A) **C** paratype SMF 32678, notopodium of chaetiger 3 showing notopodial lobe and capillaries **D** paratype SMF 32678, neuropodim of chaetiger 7 showing acicular spines and capillaries **E** paratype ZMBN 157663, notopodium of chaetiger 10 showing notopodial lobe, acicular spines, and capillaries **F** paratype SMF 32678, detail of capillary chaetae. White and black arrows in A: Lateral ciliary spots; White arrow in E: Start of crenulation in capillary chaeta. Abbreviations: Ac: acicular spines; Np: notopodial lobe. Scale bars: 100 μm (**A – C**); 40 μm (**D**); 50 μm (**E**); 10 μm (**F**).

**Figure 13.**
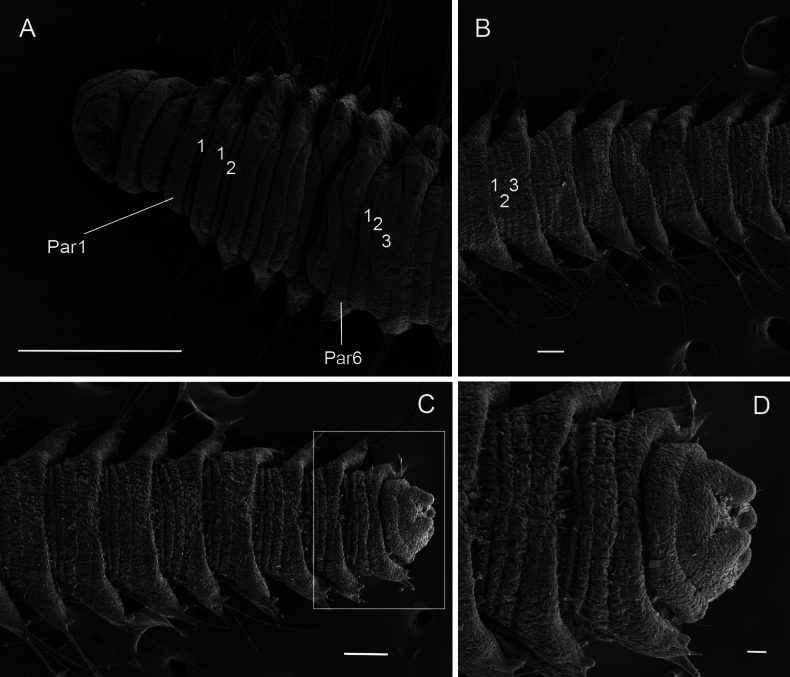
*Orbiniellapetersenae* sensu stricto, SEM**A** paratype ZMBN 157663, anterior end, ventral view showing narrow annuli between parapodia 1 and 7 **B** paratype ZMBN 157650, midbody, ventral view showing narrow annuli between parapodia 17 and 24 **C** paratype ZMBN 157650, posterior end, ventral view **D** paratype ZMBN 157650, detail of last two chaetigers together with pygidium (insert of C). Numbers indicate narrow annuli between parapodia. Abbreviations: Par1: first parapodium; Par6: sixth parapodium. Scale bars: 500 μm (**A**); 100 μm (**B, C**); 20 μm (**D**).

The prostomium shape varied between elongate (Fig. 12A) and broad (Fig. 13A), both with a rounded anterior margin (Table 1). In some specimens from the *O.petersenae* sensu lato type series material, brownish eyespots with poorly defined borders were observed. Most of the paratypes showed the same type of peristomium as in the holotype (i.e., the first ring shorter than the second one), but a few paratypes were with equal rings. Notopodia varied from digitate, long, and thin (Fig. 12C) to digitate, short and thick (Fig. 12E). Some of the IceAGE specimens presented the same three patterns of dorsal segmental pigmentation observed in *O.parapari*, possibly due to better preservation conditions.

SEM micrographs showed one single prominent lateral ciliary congregation at each side of the prostomium, which we interpret as nuchal organs (Fig. 12B). Capillary chaetae with crenulation occurring on one side along the whole chaeta or along half of its length (Fig. 12D, E). The holotype and the paratypes showed capillaries longer than body width in anterior segments as stated by Parapar et al. (2015). However, some specimens from the material collected in this study presented shorter capillaries equal to body width. Acicular spines were up to five per ramus, instead of up to three reported in Parapar et al. (2015) (Fig. 12D).

As mentioned above, among the NE Atlantic/Nordic *Orbiniella* species, *Orbiniellapetersenae* sensu stricto is most similar to *O.parapari* sp. nov., furthermore, these two species have an overlapping distribution. As in the case of *O.parapari* sp. nov., *O.petersenae* sensu stricto shares a number of morphological characters with the seven deep-sea congeners: *O.andeepia*, *O.longilobata*, *O.rugosa*, *O.tumida*, *O.abyssalis*, *O.armata*, and *O.mimica* (see remarks section of *O.parapari* sp. nov. for comparison of the two species). One sample from a station near Jan Mayen (1243 m) contained an outstanding number of *O.petersenae* sensu stricto (i.e., 322 specimens).

##### Distribution.

Iceland Sea and southern Greenland Sea, 1053–2407 m. Possibly Norwegian Sea, 2525–3892 m (Fig. 11).

#### ﻿ Orbiniella

Taxon classificationAnimaliaOrbiniidaOrbiniidae

sp.

98986F42-B188-522F-A71F-13DBA7D0CF67

Figs 14 , 15 , 16

Orbiniella petersenae : Parapar et al. 2015: 333–343, figs 3–9 (in part).

##### Clade.

Deep 4.

##### Material examined.

ZMBN 130943 (1 spm); ZMBN 157696 (1 spm on SEM stub); ZMBN 157665 (5 spms); ZMBN 157666 (2 spms on SEM stub); ZMBN 157667 (1 spm on SEM stub, DNA voucher Orbi47). 16 specimens from the *O.petersenae* sensu lato type series: IINH 43198 (5 spms). IINH 43231 (11 spms).

##### Description of ethanol preserved specimens.

DNA voucher Orbi47 (ZMBN 157667) incomplete with 15 chaetigers, 1.7 mm long and 0.3 mm wide at level of chaetiger 6. Body short and thick, with uniform width. Two more specimens from the same sample as the DNA voucher (ZMBN 130943 and ZMBN 157696) in poor condition (i.e., anterior fragments of fewer than ten segments and destroyed chaetae). Pigmentation lacking in all analysed specimens.

**Figure 14.**
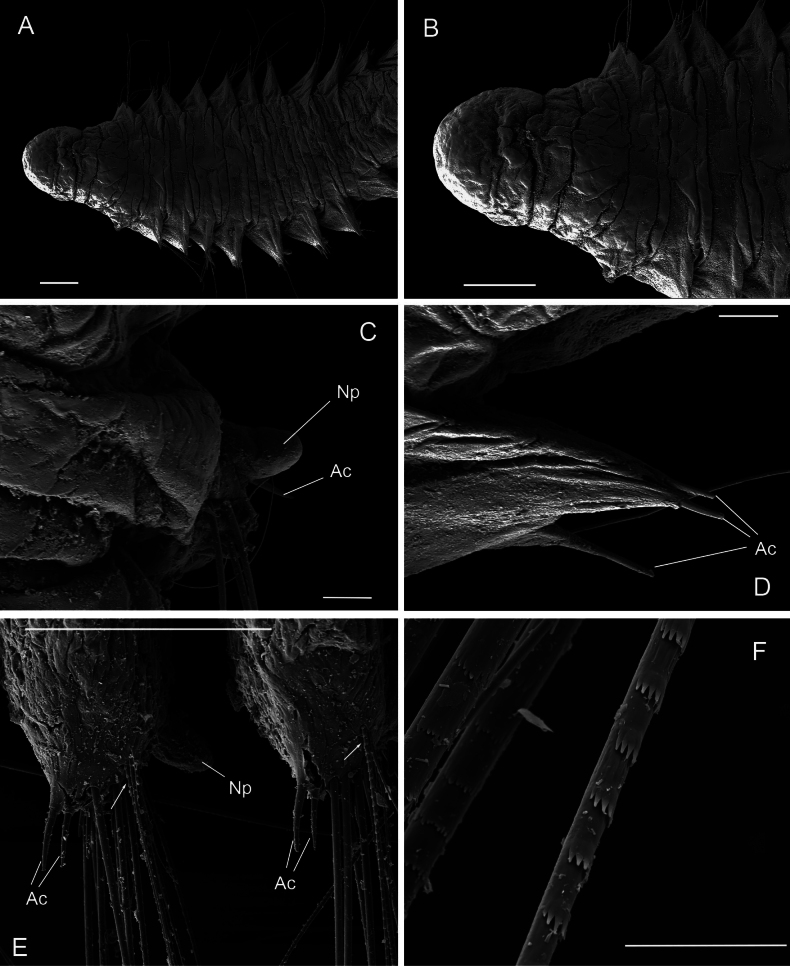
*Orbiniella* sp. SEM**A** specimen ZMBN 157696, anterior end, ventral view **B** specimen ZMBN 157696, detail of prostomium and peristomium **C** specimen ZMBN 157667, notopodium of chaetiger 6 showing notopodial lobe, acicular spine, and capillaries **D** specimen ZMBN 157696 Neuropodium of chaetiger 10 showing acicular spines **E** specimen ZMBN 157666, notopodia of chaetigers 5 and 6 showing notopodial lobe, acicular spines, and capillaries **F** specimen ZMBN 157666, detail of capillary chaetae. White arrows in E: Start of crenulation in capillary chaeta. Abbreviations: Ac: acicular spines; Np: notopodial lobe. Scales: 100 μm (**A, B, E**); 20 μm (**C, D**); 10 μm (**F**).

Prostomium broad with rounded anterior margin, without eyespots (Fig. 14A). SEM micrographs showed no cilia on prostomium. Peristomium with two prominent achaetous segments, first segment shorter than second segment, distinctly separated from each other and first chaetiger by narrow annulus (Fig. 14B). Segmental annulation consisted of two narrow annuli between parapodia from parapodium 1 until end of available fragment (Fig. 14A). Posterior part and pygidium not observed.

Parapodia biramous, triangular-like, of similar size throughout fragment. Posterior notopodial lobes from chaetiger 1, digitate, short, and thick (Fig. 14C). Posterior neuropodial lobes absent. Crenulated capillary chaetae and acicular spines in both rami from chaetiger 1. Capillaries in anterior segments longer than body width and numerous (7–10 per bundle), shorter and reduced in number in posterior segments. Acicular spines short, stout, and smooth, up to three per ramus (Fig. 14C, D).

##### Description of formalin preserved specimens.

Five well preserved specimens originally fixed in formalin collected in the Norwegian Sea (ZMBN 157665) as well as 16 specimens from *O.petersenae* sensu lato type series from the Iceland Sea shared similar morphology with the DNA voucher from Deep 4 clade (i.e., same segmental annulation pattern and chaetal distribution type). A morphological description of these specimens is provided below, although the affinity of them to Deep 4 clade remains unconfirmed with molecular data.

**Figure 15.**
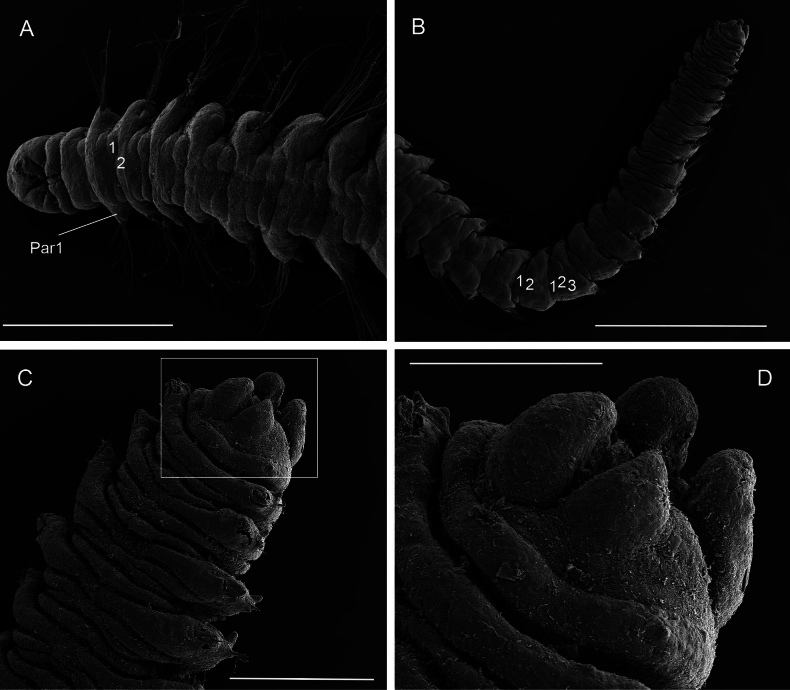
*Orbiniella* sp. SEM of ZMBN 157666 **A** anterior end, ventral view showing narrow annuli between parapodia 1 and 8 **B** midbody and posterior end, ventral view showing narrow annuli between parapodia 9 and 29 **C** posterior end, dorsal view **D** Detail of pygidium (insert of C). Numbers indicate narrow annuli between parapodia. Abbreviation: Par1: first parapodium. Scale bars: 500 μm (**A**); 1000 μm (**B**); 200 μm (**C**); 100 μm (**D**).

Shape of prostomium varies between broad and elongate, with rounded anterior margin (Fig. 15A). Eyespots absent. Peristomium with two prominent achaetous segments, first segment shorter than second segment, distinctly separated from each other and first chaetiger by narrow annulus.

Segmental annulation pattern as follows: two narrow annuli between parapodia from parapodium 1 until posterior body and three narrow annuli between parapodia in posterior-most body (Fig. 15A, B). Annulation well defined in pre-pygidial region (Fig. 15C). From chaetiger 7–10 onwards segments becoming longer, more square-shaped.

Parapodia biramous, triangular-like, of similar size throughout body. Postchaetal notopodial lobes from chaetiger 1, digitate, short, and thick (Fig. 14C, E). Postchaetal neuropodial lobes absent. Crenulated capillary chaetae and acicular spines in both rami from chaetiger 1. Notopodia and neuropodia bearing each 7–10 long crenulated capillaries, being longer than body width in anterior segments and shorter and less numerous in posterior segments. Capillary chaetae with crenulation occurring on one side along whole chaeta or along half of its length (Fig. 14E). Last five or six posterior segments slightly shorter and last two achaetous (Fig. 15C). Pygidium with four long and wide anal lobes (Fig. 15D).

##### Distribution.

**Figure 16.**
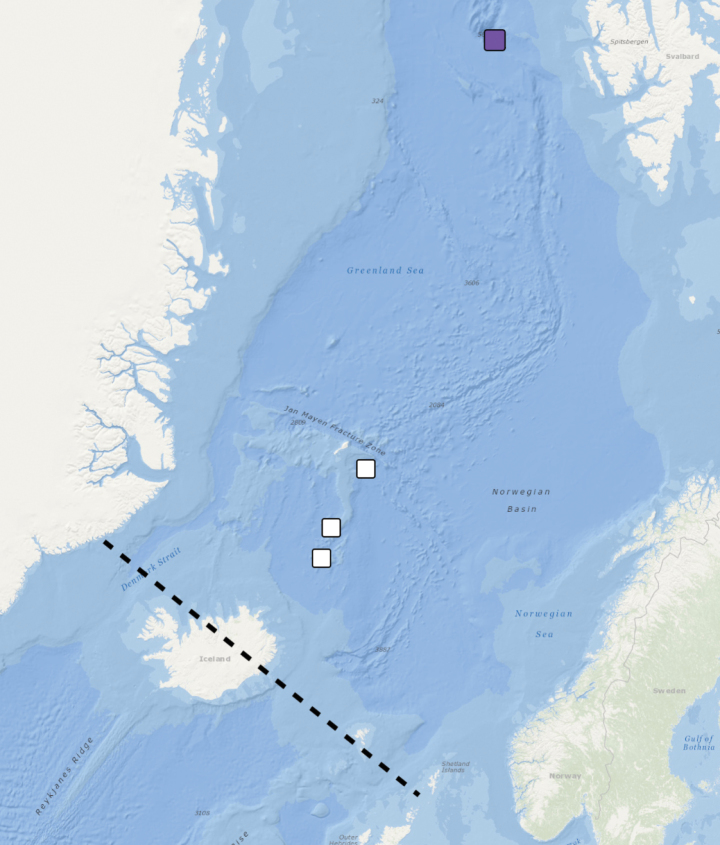
Distribution of *Orbiniella* sp. Black lines – Greenland-Scotland Ridge (GSR), coloured square – station with examined morphology and molecular data, white squares – stations with examined morphology.

Northern Greenland Sea, 3356 m. Possibly Iceland Sea and Norwegian Sea, 1844–2525 m (Fig. 16).

## ﻿Discussion

### ﻿Systematics and species delimitation

*Orbiniellapetersenae*, the only species of *Orbiniella* known up to date from the North Atlantic, was recorded from a great diversity of bottom water temperatures and depths between different localities. Due to this environmental plasticity and a limited number of morphological characters used in *Orbiniella* diagnoses, we assessed if more than one species is present in the area using molecular tools. In the present study, we report four *Orbiniella* species from the Nordic Seas and one more species from the deep waters south of Iceland based on the combination of molecular and morphological data utilised in the phylogenetic and species delimitation analyses. We restrict the species name *Orbiniellapetersenae* to one of the genetic lineages limiting its geographical and vertical ranges to the deep-sea areas of the Iceland Sea and the southern Greenland Sea. Three more species, *O.griegi* sp. nov. (shallow habitats along the Greenland-Scotland Ridge and along the Norwegian coast), *O.parapari* sp. nov. (deep Nordic Seas), and *O.mayhemi* sp. nov. (deep NE Atlantic, south of Iceland), are here formally described as new taxa. One more species, reported in the northern deep Greenland Sea and possibly also in the deep Iceland Sea and the Norwegian Sea, is described here but not formally named due to lack of specimens allowing more detailed morphological studies and further molecular information

*Orbiniellapetersenae* sensu stricto, *O.parapari* sp. nov., and *O.griegi* sp. nov. show a unique segmental annulation pattern in the genus, combining one, two, and three narrow annuli between parapodia. Moreover, the former two species display the highest number of acicular spines (up to five in *O.petersenae* sensu stricto and up to six in *O.parapari* sp. nov.) in the parapodia within the whole genus. *Orbiniellagriegi* sp. nov. has a unique pygidium with four anal lobes assembled together (Fig. 2).

Up to date, the only available genetic data on *Orbiniella* were the four 16S sequences belonging to *Orbiniella sp.* 49 and *Orbiniella sp.* 279 from the NE Pacific in Bonifácio et al. (2020) and the Nad4, COI, 16S and 18S sequences of *O.plumisetosa* in Bleidorn (2005) and Bleidorn et al. (2009). Georgieva et al. (2023) reported two new *Orbiniella* species (i.e., *Orbiniellajamesi* Georgieva, Wiklund, Ramos, Neal, Glasby & Gunton, 2023 and *Orbiniella* sp.) from the New South Wales (Australia) based on COI, 16S and 18S sequences. These species were not included in the present analysis. We do not consider them belonging to *Orbiniella* due to the presence of branchiae, a character not reported in any other *Orbiniella* species (Blake 2017, 2020, 2021). Furthermore, both species were placed within the *Scoloplos*/*Leitoscoloplos* clade in our preliminary analysis of orbiniid phylogeny based on mitochondrial genome and nuclear data (in preparation) and their generic affinity requires further clarification. Our data include sequences of three molecular markers (COI, 16S and ITS2) of five *Orbiniella* species from the NE Atlantic and the Nordic seas considerably expanding the taxon and marker coverage in this poorly studied orbiniid genus.

*Orbiniella* was not recovered as monophyletic in our phylogenetic analyses based on the combined dataset. *Orbiniellaplumisetosa* was sister to *Phylonorvegicus*, with high support only in the concatenated BI, while all other *Orbiniella* species were combined into another highly supported clade. This can be due to the amount of missing data in the analysis both in taxon and sequence data coverage. Nevertheless, the Orbiniidae systematics remains unresolved and the boundaries of most of the genera are poorly understood (Bleidorn et al. 2009; Zhadan et al. 2015; Meca et al. 2021). Further investigation of the composition and the placement of *Orbiniella* within the orbiniid tree can clarify the monophyletic status of the genus.

Five well supported clades within *Orbiniella* and three more species present as single individuals were recovered in our phylogenetic analyses. The species delimitation analyses recovered eight putative species. Although COI data were absent in *O.parapari* sp. nov. and 16S data were absent in *Orbiniella* sp., all five species had at least one mitochondrial and one nuclear marker in the combined dataset, supporting robust phylogeny and species delimitation results. Two delimitation conflicts were reported in the analyses. For the COI dataset, both PTP and ASAP analyses supported splitting *O.griegi* sp. nov. into two putative species. For the ITS2 dataset, the ASAP analyses supported combining *O.mayhemi* and *Orbiniella* sp. into a single putative species. All other analyses delimited the five North Atlantic/Nordic clades as separate species. Additionally, we found several consistent morphological characters in each of the putative species supporting the overall proposed delimitation scheme (Table 1).

The four new species of *Orbiniella* described in this study formed a highly supported clade in the combined and in the individual marker datasets. The uncorrected p-distances between the four species in this clade ranged from 17.9 to 29.7% in COI, from 6.6 to 22.2% in 16S, and from 2.1 to 30.6% in ITS2. *Orbiniellapetersenae* sensu stricto was genetically the most distant species form the rest of the species found in the NE Atlantic and the Nordic seas differing by more than 20% in p-distance in COI, 16S and ITS2 markers.

### ﻿Morphological diagnostic characters

Blake (2020) reviewed all the morphological characters used for species description in the deep-water *Orbiniella* and listed seven key characters required to separate them. Among those, the ones which better discriminated the NE Atlantic/Nordic *Orbiniella* species were: (1) the segmental annulation pattern, (2) number and shape of the acicular spines and (3) shape and length of the anal lobes. The shape of the prostomium, relative length of the peristomial segments and shape and length of the notopodia showed a great intraspecific variation within *O.petersenae* sensu stricto and *O.parapari* sp. nov. (see species descriptions and Table 1). Parapar et al. (2015) questioned the taxonomic value of the intersegmental ring pattern since the annulation was more conspicuous under SEM and thus could be an artefact of the critical point drying. We evaluated the appropriateness of SEM procedures for the analysis of the narrow annuli between parapodia in the NE Atlantic/Nordic *Orbiniella* through applying methylene blue staining in the studied material, and we could confirm the annulation pattern being identical in the stained wet specimens and the specimens studied for SEM. Therefore, we reaffirm the use of the segmental annulation pattern for species discrimination in *Orbiniella* and the application of both SEM and light microscopy in combination with methylene blue staining for the analysis of this character.

We also explored the taxonomic utility of the dorsal segmental pigmentation, characteristics of the crenulation in the capillaries, the presence of the nuchal organs and the eyes. Dorsal segmental pigmentation was discovered in two species: *O.petersenae* sensu stricto and *O.parapari* sp. nov.; however, it was not specific for either of them nor was it present in every examined specimen. Therefore, we do not consider dorsal segmental pigmentation to be a character useful for species discrimination. We suggest variation in pigmentation might be an artefact of fixation and conservation procedures, and observations of pigmentation in live specimens should help to clarify the use of it in species diagnoses. SEM observations allowed evaluating the variation in (1) the start of the crenulation and (2) the pronunciation of this crenulation in the capillaries of some specimens. The crenulation can start from the base or from the middle of a capillary chaeta, and it can be pronounced from the start or be weak basally and more obvious distally. All kind of capillaries were present in the five NE Atlantic/Nordic *Orbiniella* species and, therefore, it does not appear to be a useful character for species differentiation. SEM micrographs allowed us to elucidate some differences in the nuchal organs in *Orbiniella* species. *Orbiniellagriegi* sp. nov. presented two lateral, not very prominent ciliary spots, whilst *O.petersenae* sensu stricto and *O.parapari* sp. nov. showed one single prominent lateral ciliary congregation on each side of the prostomium. Although no nuchal organs were found in *O.mayhemi* sp. nov. or in *Orbiniella* sp., more observations of specimens are needed to confirm the absence of the nuchal organs in these species. The fact that just a small portion of all the observed specimens of *O.petersenae* sensu stricto and *O.parapari* sp. nov. showed eyespots, indicates that their absence might be due to fading during long-term storage in ethanol. We consider the presence of eyes a character not reliable for species discrimination. We cannot discard the presence of eyespots in *O.griegi* sp. nov., *O.mayhemi* sp. nov. and *Orbiniella* sp., as well as in other *Orbiniella* species seemingly lacking eyes. Similar to the dorsal pigmentation, observations of live specimens should help in clarifying the use of eyes in species diagnoses.

### ﻿Species distribution

Our data suggest that the species of *Orbiniella* in the NE Atlantic and adjacent Nordic Seas have restricted vertical distribution either in the shelf and upper slope habitats as in *O.griegi* sp. nov. distributed across the Norwegian shelf and the Greenland-Scotland Ridge (GSR) between 171–781 m, or at the depths below 1000 m as in *O.petersenae* sensu stricto, *O.parapari*, *O.mayhemi*, and *Orbiniella* sp. Notably, all deep-sea *Orbiniella* species reported in this study occur on either side of the GSR only: *O.petersenae* sensu stricto and *O.parapari* sp. nov. were reported in the Iceland Sea and Norwegian Sea, and also in the southern Greenland Sea, *Orbiniella* sp. in the northern Greenland Sea and possibly also in the Iceland Sea and Norwegian Sea, whilst *O.mayhemi* inhabits the waters south-west of Iceland. Despite low number of species analysed here, the GSR with its maximum depth at ca. 900 m appears to act as a barrier for the deep-sea *Orbiniella* species dispersal. This corroborates the preliminary results on other annelid genera (Budaeva et al. 2019), on isopods (Brix and Svavarsson 2010; Schnurr et al. 2018) and on cumaceans (Uhlir et al. 2021). Notably, GSR coincides with the biogeographical border drawn for the shelf fauna separating the Arctic and the Boreal regions (Mironov 2013). In the eastern part of the North Atlantic this border extends along the Norwegian shelf up to the Barents Sea with coastal Norwegian shelf areas warmed by the Gulf Stream and inholding Atlantic fauna. The significance of this border for the deep-sea species distribution has not been clearly demonstrated using comprehensive data. Our data on *Orbiniella* species distribution support the presence of the border in both the upper and lower bathyal areas.

## ﻿Conclusions

Diversity of *Orbiniella* in the NE Atlantic and the adjacent Nordic Seas constitutes at least five species, four of which are new to science. Three species, *Orbiniellaparapari* Meca & Budaeva, sp. nov., *Orbiniellagriegi* Meca & Budaeva, sp. nov., and *Orbiniellamayhemi* Meca & Budaeva, sp. nov., are formally described and the fourth species is left unnamed due to the scarcity of the available material. The diagnosis and distribution of *Orbiniellapetersenae*, the only species previously known from the region, are clarified. All five species are morphologically very similar but can be separated by subtle differences in their pattern of segmental annulation, number and shape of the acicular spines, and length and shape of the four anal lobes in the pygidium, highlighting the taxonomic value of these characters for species discrimination in *Orbiniella*.

The Greenland-Iceland-Scotland Ridge acts as a barrier for the deep-sea *Orbiniella* species distribution in the North Atlantic and the Nordic Seas, with three of the deep-water species (i.e., *O.petersenae* sensu stricto, *O.parapari* sp. nov., and *Orbiniella* sp. reported exclusively north of the ridge in the cold Arctic waters, and *O.mayhemi* sp. nov. reported south of Iceland in warmer Atlantic waters. The shallow water *O.griegi* sp. nov. is distributed along the biogeographical border between the North Atlantic and the Arctic regions.

## Supplementary Material

XML Treatment for Orbiniella

XML Treatment for Orbiniella griegi

XML Treatment for Orbiniella mayhemi

XML Treatment for Orbiniella parapari

XML Treatment for Orbiniella petersenae

XML Treatment for Orbiniella

## ﻿Additional information

### Author ORCIDs

Miguel A. Meca https://orcid.org/0000-0002-3798-6809

Katrine Kongshavn https://orcid.org/0000-0002-4265-0912

Nataliya Budaeva https://orcid.org/0000-0001-9748-2285

### Data availability

All of the data that support the findings of this study are available in the main text or Supplementary Information.

## Supplementary materials

Supplementary material 1 Sampling data, PCR protocol and genetic distances Data type xlsx Explanation note **table S1.** List of material used in this study with sampling information, museum numbers, voucher numbers, GenBank accession. **table S2.** Primers and PCR settings for COI, 16S rDNA and ITS2. **table S3.** Pairwise genetic distances within and between the NE Atlantic/Nordic clades and public *Orbiniella* sequences. https://binary.pensoft.net/file/1074857This dataset is made available under the Open Database License (http://opendatacommons.org/licenses/odbl/1.0/). The Open Database License (ODbL) is a license agreement intended to allow users to freely share, modify, and use this Dataset while maintaining this same freedom for others, provided that the original source and author(s) are credited.Miguel A. Meca, Jon Anders Kongsrud, Katrine Kongshavn, Tom Alvestad, Karin Meißner, Nataliya Budaeva

Supplementary material 2 Phylogenetic trees regarding COI, 16 and ITS2 markers, and species delimitation results Data type docx Explanation note **fig. S1.** Maximum likelihood (ML) analysis based on the concatenated dataset of COI, 16S and ITS2. Bootstrap values are shown on the nodes. Capital letters correspond with the clades discussed in the text. **fig. S2.** Bayesian inference (BI) and Maximum likelihood (ML) analysis based on COI gene. Node support is indicated on the nodes (PP/BS). Capital letters correspond with the clades discussed in the text. **fig. S3.** Bayesian inference (BI) and Maximum Likelihood (ML) analysis based on ITS2 gene. Node support is indicated on the nodes (PP/BS). Capital letters correspond with the clades discussed in the text. **fig. S4.** Maximum likelihood (ML) analysis based on 16S gene. Bootstrap values are shown on the nodes. Capital letters correspond with the clades discussed in the text. **fig. S5.** Bayesian inference (BI) based on 16S gene. Bayesian posterior probabilities are shown on the nodes. Capital letters correspond with the clades discussed in the text. **File S1.**PTP species delimitation. A. COI. B. 16S. C. ITS2. **A.** Species delimitation – COI fragment: PTP results; **B.** Species delimitation – 16S fragment: PTP results; **C.** Species delimitation – ITS2 fragment: PTP results; **File S2.**ASAP species delimitation. A. COI. B. COI. C. ITS2. **A.** Species delimitation – COI fragment: ASAP results; **B.** Species delimitation – 16S fragment: ASAP results; **C.** Species delimitation – ITS2 fragment: ASAP results https://binary.pensoft.net/file/1074858This dataset is made available under the Open Database License (http://opendatacommons.org/licenses/odbl/1.0/). The Open Database License (ODbL) is a license agreement intended to allow users to freely share, modify, and use this Dataset while maintaining this same freedom for others, provided that the original source and author(s) are credited.Miguel A. Meca, Jon Anders Kongsrud, Katrine Kongshavn, Tom Alvestad, Karin Meißner, Nataliya Budaeva
