# Supplementary material for: ﻿Diversity of Orbiniella (Orbiniidae, Annelida) in the North Atlantic and the Arctic
Source: Zookeys. 2024 Jun 20;1205:51–88. doi: 10.3897/zookeys.1205.120300 (PMC11211660; doi:10.3897/zookeys.1205.120300)
Supplement: Supplementary material 2 — Phylogenetic trees regarding COI, 16 and ITS2 markers, and species delimitation results [file zookeys-1205-051_article-120300__-s002.docx]

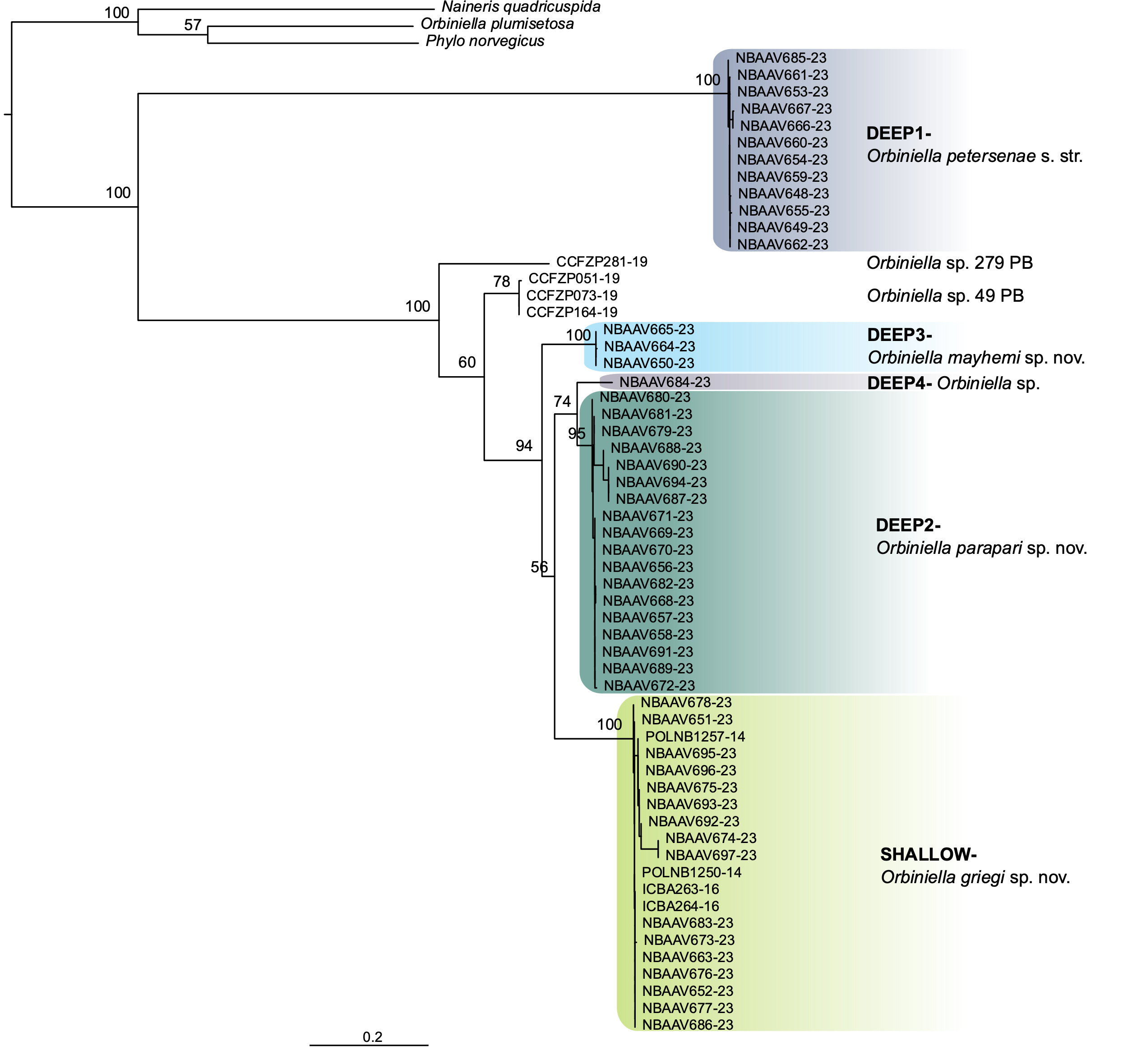


**Fig. S1.** Maximum likelihood (ML) analysis based on the concatenated dataset of COI, 16S and ITS2. Bootstrap values are shown on the nodes. Capital letters correspond with the clades discussed in the text.

**
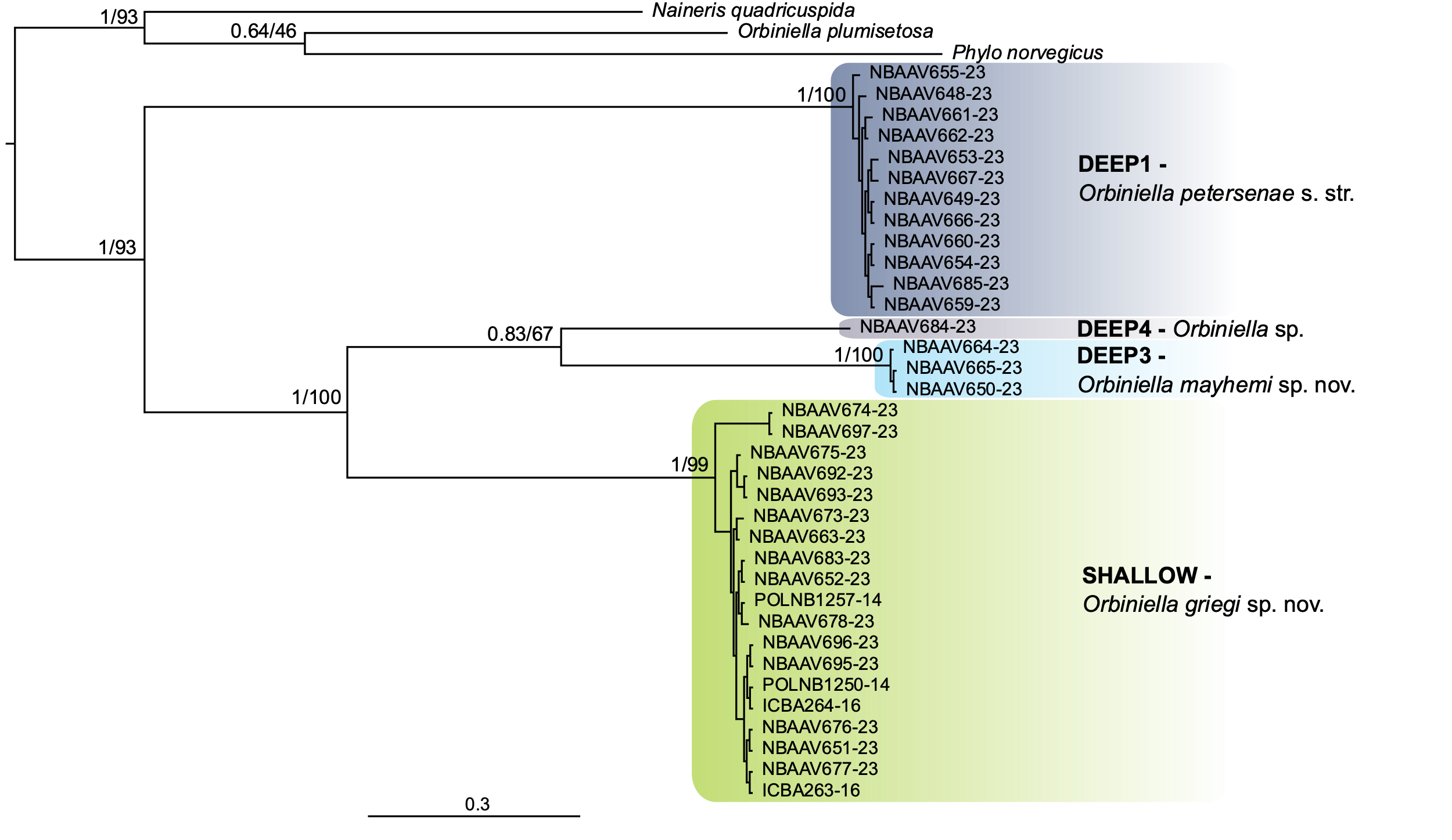
**

**Fig. S2.** Bayesian inference (BI) and Maximum likelihood (ML) analysis based on COI gene. Statistic support is indicated on the nodes (BI/ML). Capital letters correspond with the clades discussed in the text.


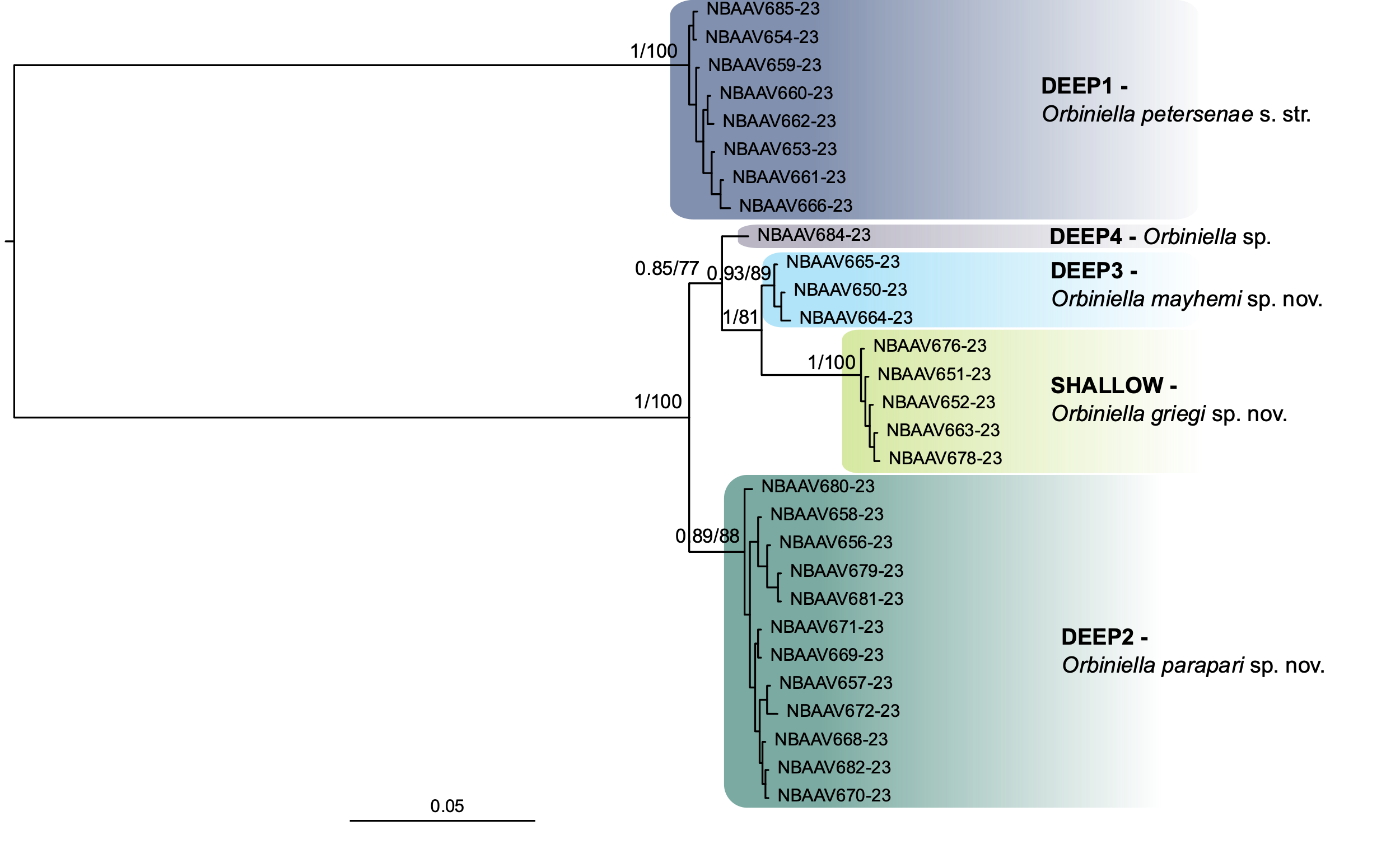


**Fig. S3.** Bayesian inference (BI) and Maximum Likelihood (ML) analysis based on ITS2 gene. Statistic support is indicated on the nodes (PP/BS). Capital letters correspond with the clades discussed in the text.

**
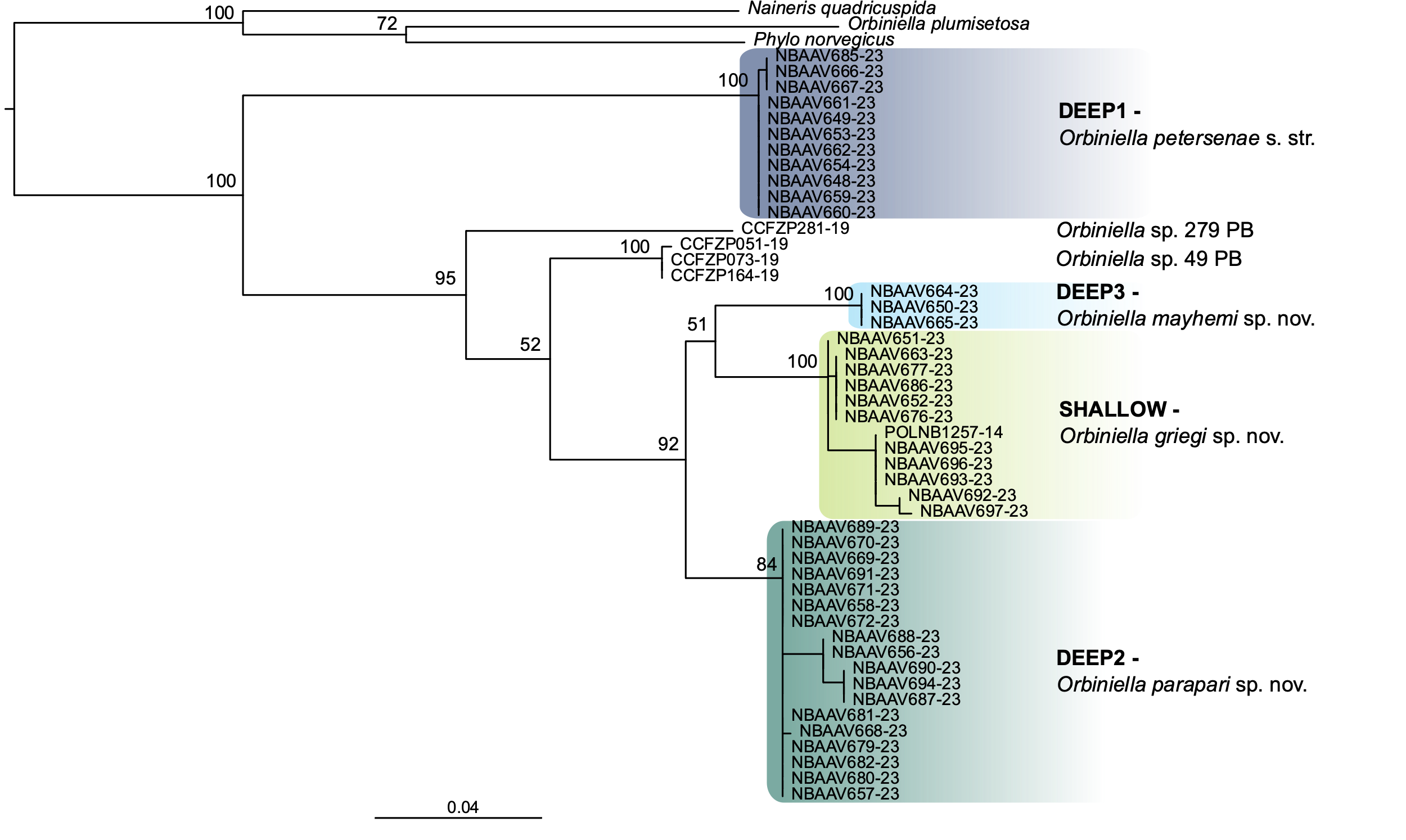
**

**Fig. S4.** Maximum likelihood (ML) analysis based on 16S gene. Bootstrap values are shown on the nodes. Capital letters correspond with the clades discussed in the text.

**
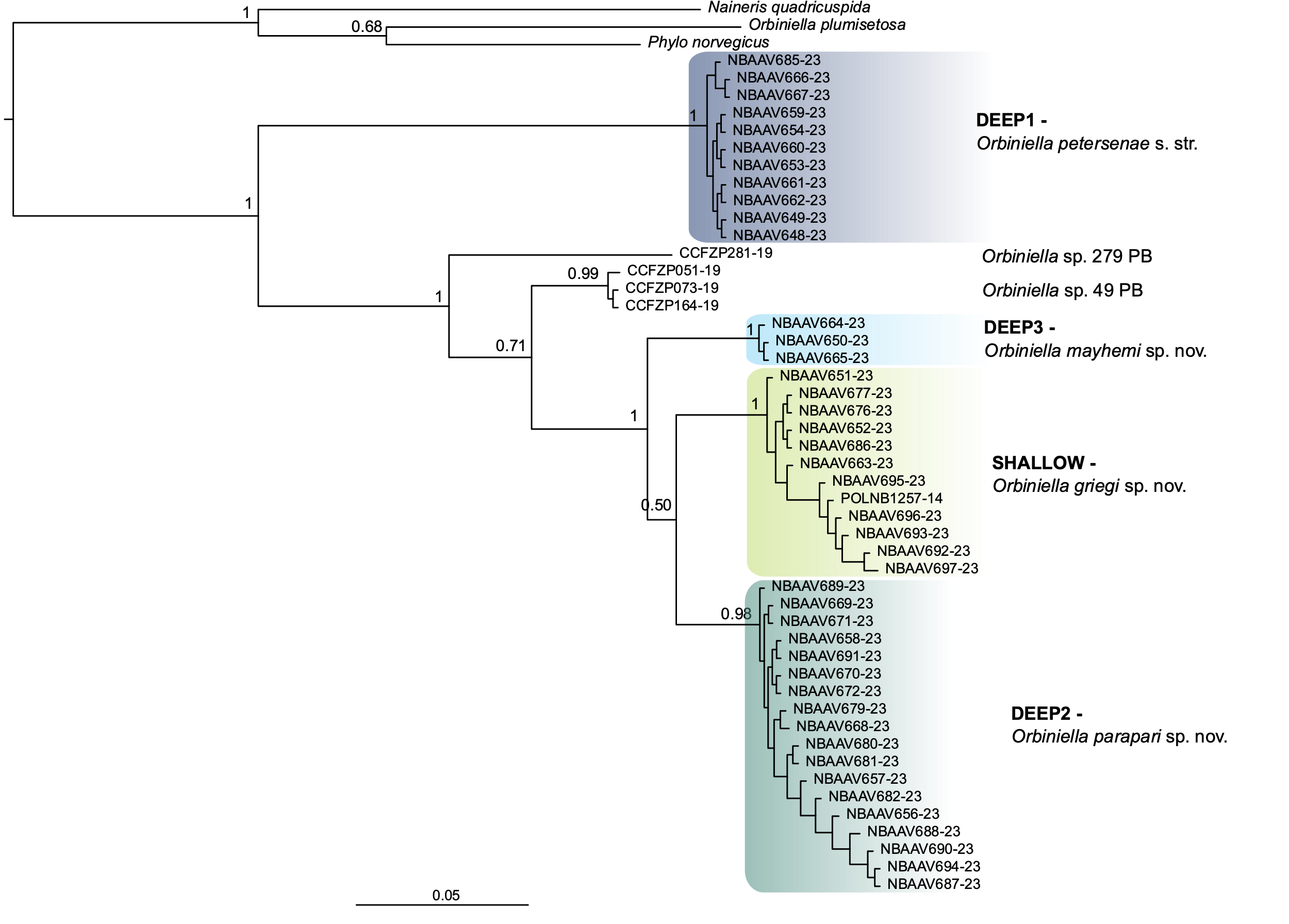
**

**Fig. S5.** Bayesian inference (BI) based on 16S gene. Bayesian posterior probabilities are shown on the nodes. Capital letters correspond with the clades discussed in the text.

**File S1.** PTP species delimitation. A. COI. B. 16S. C. ITS2. D. Combined data set

**A. Species delimitation – COI fragment: PTP results**

**Results based on the Maximum Likelihood reconstruction**

**Maximum Likelihood tree**


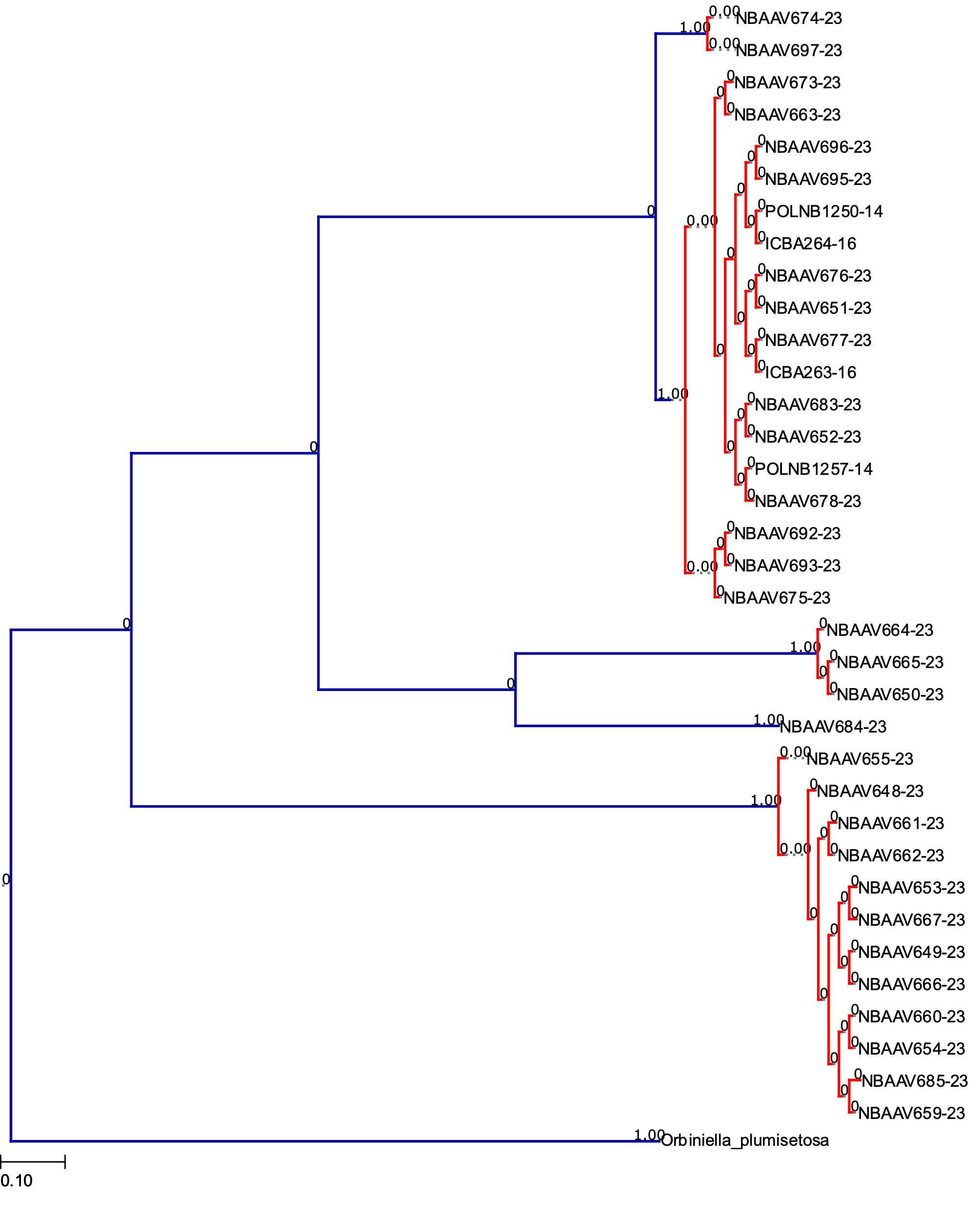


**Maximum Likelihood partition**

*Orbiniella plumisetosa* (support = 1.000)

Orbiniella_plumisetosa

*Orbiniella petersenae sensu stricto* (support = 0.990)

NBAAV685-23,NBAAV659-23,NBAAV655-23,NBAAV648-23,NBAAV661-23,NBAAV662-23,NBAAV653-23,NBAAV667-23,NBAAV649-23,NBAAV666-23,NBAAV660-23,NBAAV654-23

*Orbiniella mayhemi* sp. nov. (support = 0.999)

NBAAV664-23,NBAAV665-23,NBAAV650-23

*Orbiniella* sp. (support = 1.000)

NBAAV684-23

*Orbiniella griegi* sp. nov. (support = 0.998)

NBAAV674-23,NBAAV697-23

*Orbiniella griegi* sp. nov. (support = 0.998)

NBAAV673-23,NBAAV663-23,NBAAV696-23,NBAAV695-23,POLNB1250-14,ICBA264-16,NBAAV676-23,NBAAV651-23,NBAAV677-23,ICBA263-16,NBAAV683-23,NBAAV652-23,POLNB1257-14,NBAAV678-23,NBAAV692-23,NBAAV693-23,NBAAV675-23

**Results based on the Bayesian inference reconstruction**

**Bayesian inference tree**


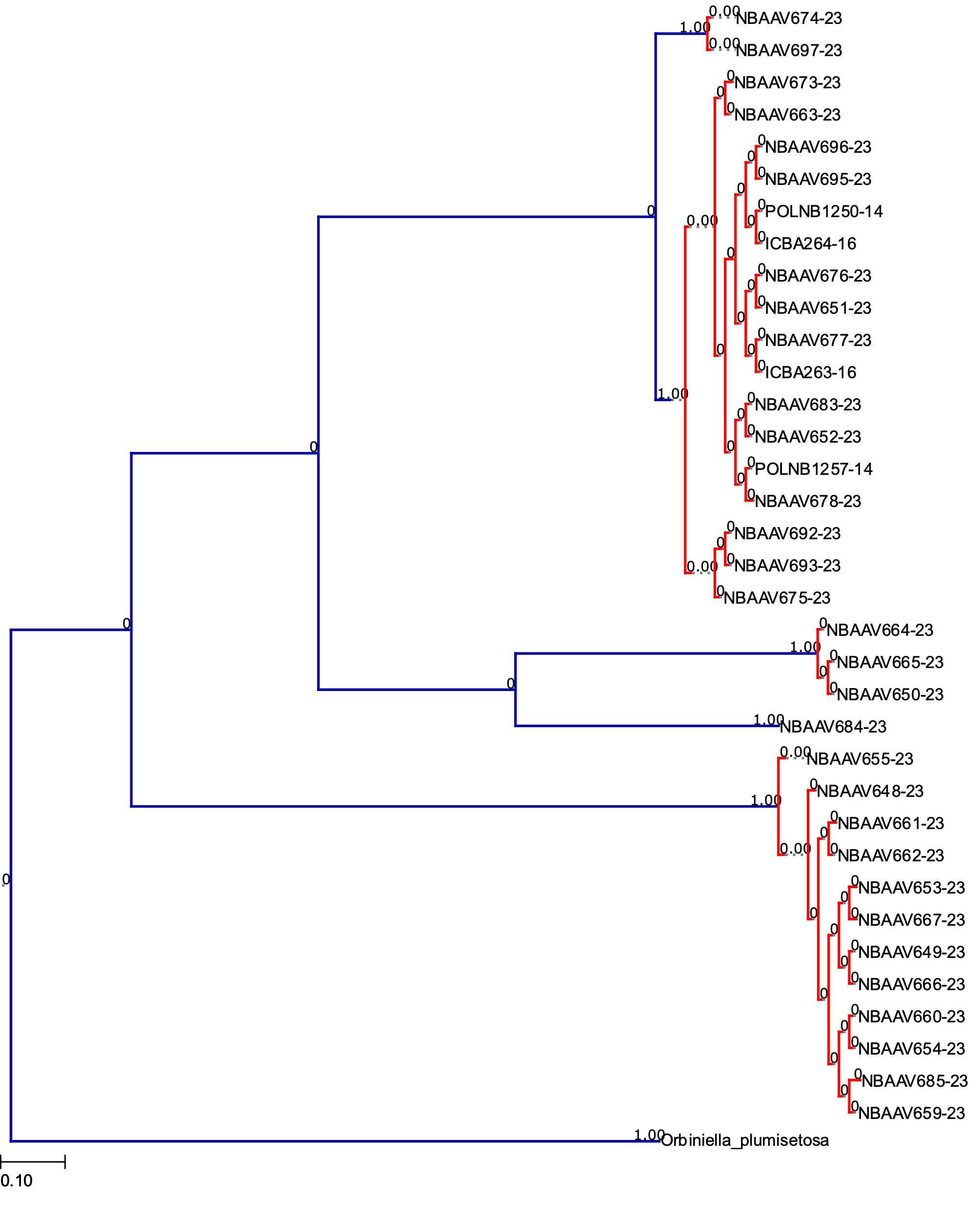


**Most supported partition found by simple heuristic search**

*Orbiniella plumisetosa* (support = 1.000)

Orbiniella_plumisetosa

*Orbiniella petersenae sensu stricto* (support = 0.990)

NBAAV685-23,NBAAV659-23,NBAAV655-23,NBAAV648-23,NBAAV661-23,NBAAV662-23,NBAAV653-23,NBAAV667-23,NBAAV649-23,NBAAV666-23,NBAAV660-23,NBAAV654-23

*Orbiniella mayhemi* sp. nov. (support = 0.999)

NBAAV664-23,NBAAV665-23,NBAAV650-23

*Orbiniella* sp. (support = 1.000)

NBAAV684-23

*Orbiniella griegi* sp. nov. (support = 0.998)

NBAAV674-23,NBAAV697-23

*Orbiniella griegi* sp. nov. (support = 0.998)

NBAAV673-23,NBAAV663-23,NBAAV696-23,NBAAV695-23,POLNB1250-14,ICBA264-16,NBAAV676-23,NBAAV651-23,NBAAV677-23,ICBA263-16,NBAAV683-23,NBAAV652-23,POLNB1257-14,NBAAV678-23,NBAAV692-23,NBAAV693-23,NBAAV675-23

**B. Species delimitation – 16S fragment: PTP results**

**Results based on the Maximum Likelihood reconstruction**

**Maximum Likelihood tree**


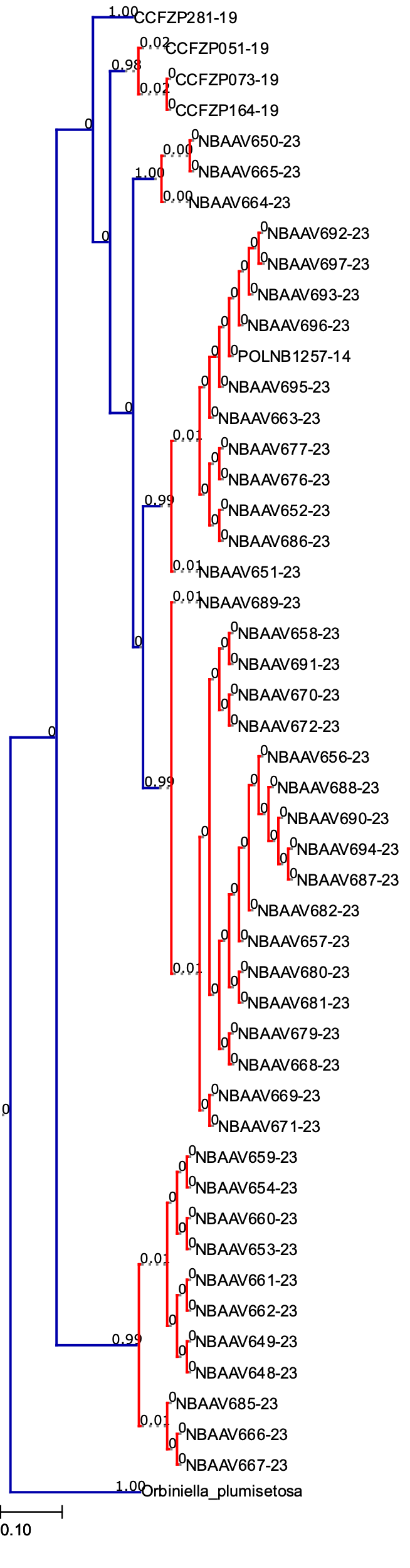


**Maximum Likelihood partition**

*Orbiniella plumisetosa* (support = 1.000)

Orbiniella_plumisetosa

*Orbiniella petersenae sensu stricto* (support = 0.988)

NBAAV666-23,NBAAV667-23,NBAAV659-23,NBAAV654-23,NBAAV660-23,NBAAV653-23,NBAAV661-23,NBAAV662-23,NBAAV649-23,NBAAV648-23,NBAAV685-23

*Orbiniella* sp. 279 PB (support = 1.000)

CCFZP281-19

*Orbiniella* sp. 49 PB (support = 0.977)

CCFZP051-19,CCFZP073-19,CCFZP164-19

*Orbiniella mayhemi* sp. nov. (support = 0.991)

NBAAV650-23,NBAAV665-23,NBAAV664-23

*Orbiniella griegi* sp. nov. (support = 0.972)

NBAAV692-23,NBAAV697-23,NBAAV693-23,NBAAV696-23,POLNB1257-14,NBAAV695-23,NBAAV663-23,NBAAV677-23,NBAAV676-23,NBAAV652-23,NBAAV686-23,NBAAV651-23

*Orbiniella parapari* sp. nov. (support = 0.989)

NBAAV689-23,NBAAV658-23,NBAAV691-23,NBAAV670-23,NBAAV672-23,NBAAV656-23,NBAAV688-23,NBAAV690-23,NBAAV694-23,NBAAV687-23,NBAAV682-23,NBAAV657-23,NBAAV680-23,NBAAV681-23,NBAAV679-23,NBAAV668-23,NBAAV669-23,NBAAV671-23

**Results based on the Bayesian inference reconstruction**

**Bayesian inference tree**

**
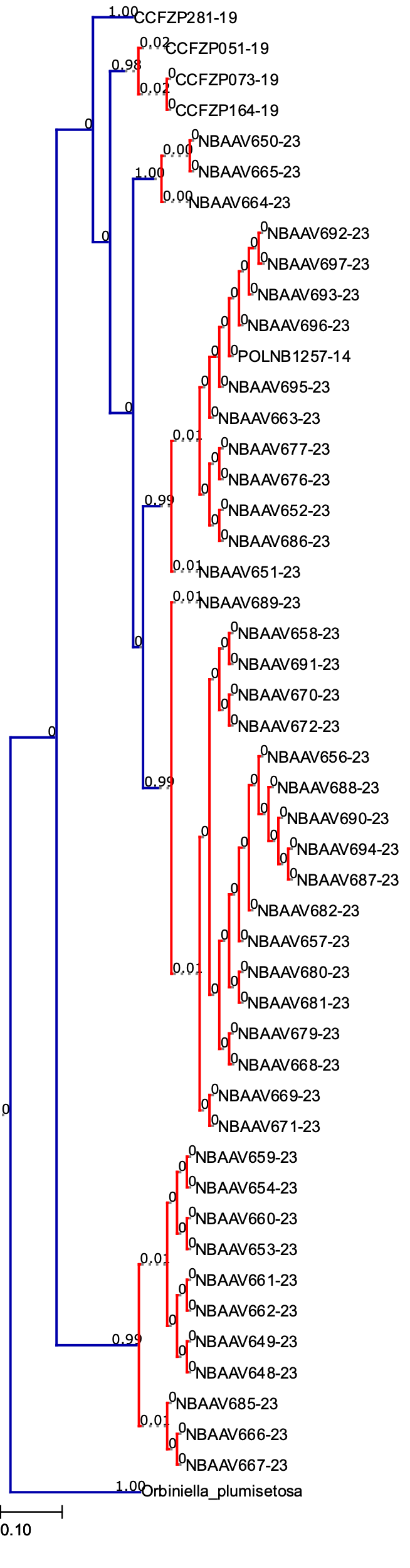
**

**Most supported partition found by simple heuristic search**

*Orbiniella plumisetosa* (support = 1.000)

Orbiniella_plumisetosa

*Orbiniella petersenae sensu stricto* (support = 0.988)

NBAAV666-23,NBAAV667-23,NBAAV659-23,NBAAV654-23,NBAAV660-23,NBAAV653-23,NBAAV661-23,NBAAV662-23,NBAAV649-23,NBAAV648-23,NBAAV685-23

*Orbiniella* sp. 279 PB (support = 1.000)

CCFZP281-19

*Orbiniella* sp. 49 PB (support = 0.977)

CCFZP051-19,CCFZP073-19,CCFZP164-19

*Orbiniella mayhemi* sp. nov. (support = 0.991)

NBAAV650-23,NBAAV665-23,NBAAV664-23

*Orbiniella griegi* sp. nov. (support = 0.972)

NBAAV692-23,NBAAV697-23,NBAAV693-23,NBAAV696-23,POLNB1257-14,NBAAV695-23,NBAAV663-23,NBAAV677-23,NBAAV676-23,NBAAV652-23,NBAAV686-23,NBAAV651-23

*Orbiniella parapari* sp. nov. (support = 0.989)

NBAAV689-23,NBAAV658-23,NBAAV691-23,NBAAV670-23,NBAAV672-23,NBAAV656-23,NBAAV688-23,NBAAV690-23,NBAAV694-23,NBAAV687-23,NBAAV682-23,NBAAV657-23,NBAAV680-23,NBAAV681-23,NBAAV679-23,NBAAV668-23,NBAAV669-23,NBAAV671-23

**C. Species delimitation – ITS2 fragment: PTP results**

**Results based on the Maximum Likelihood reconstruction**

**Maximum Likelihood Tree**


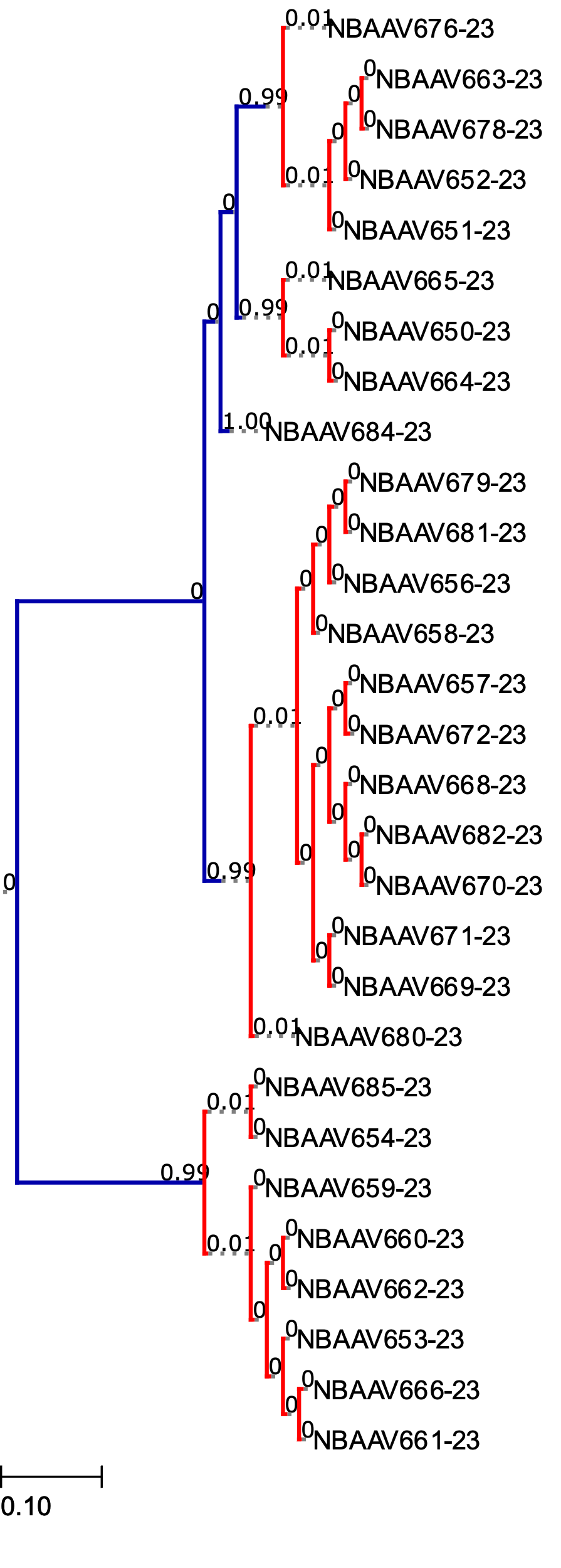


**Maximum Likelihood partition**

*Orbiniella petersenae sensu stricto* (support = 0.992)

NBAAV661-23,NBAAV666-23,NBAAV653-23,NBAAV659-23,NBAAV685-23,NBAAV654-23,NBAAV660-23,NBAAV662-23

*Orbiniella griegi* sp. nov. (support = 0.993)

NBAAV676-23,NBAAV663-23,NBAAV678-23,NBAAV652-23,NBAAV651-23

*Orbiniella mayhemi* sp. nov. (support = 0.993)

NBAAV665-23,NBAAV650-23,NBAAV664-23

*Orbiniella* sp. (support = 1.000)

NBAAV684-23

*Orbiniella parapari* sp. nov. (support = 0.991)

NBAAV679-23,NBAAV681-23,NBAAV656-23,NBAAV658-23,NBAAV657-23,NBAAV672-23,NBAAV668-23,NBAAV682-23,NBAAV670-23,NBAAV671-23,NBAAV669-23,NBAAV680-23

**Results based on the Bayesian inference reconstruction**

**Bayesian inference tree**


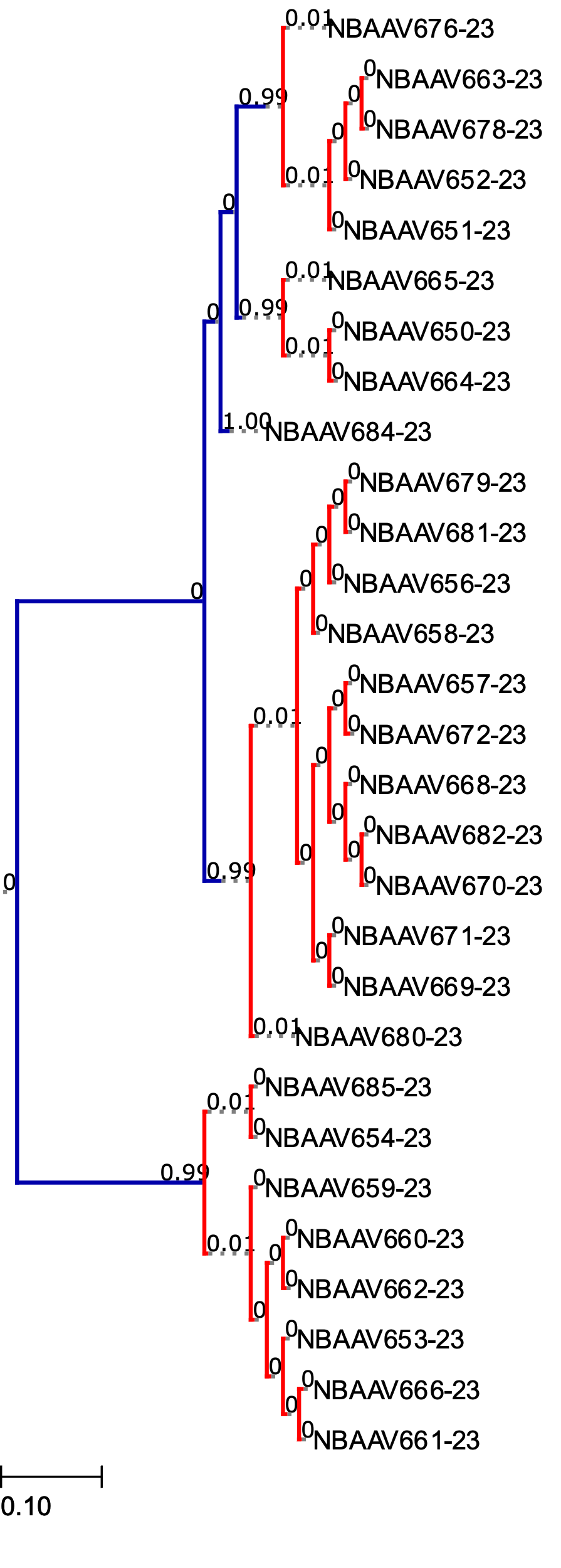


**Most supported partition found by simple heuristic search**

*Orbiniella petersenae sensu stricto* (support = 0.992)

NBAAV661-23,NBAAV666-23,NBAAV653-23,NBAAV659-23,NBAAV685-23,NBAAV654-23,NBAAV660-23,NBAAV662-23

*Orbiniella griegi* sp. nov. (support = 0.993)

NBAAV676-23,NBAAV663-23,NBAAV678-23,NBAAV652-23,NBAAV651-23

*Orbiniella mayhemi* sp. nov. (support = 0.993)

NBAAV665-23,NBAAV650-23,NBAAV664-23

*Orbiniella* sp. (support = 1.000)

NBAAV684-23

*Orbiniella parapari* sp. nov. (support = 0.991)

NBAAV679-23,NBAAV681-23,NBAAV656-23,NBAAV658-23,NBAAV657-23,NBAAV672-23,NBAAV668-23,NBAAV682-23,NBAAV670-23,NBAAV671-23,NBAAV669-23,NBAAV680-23

**File S2.** ASAP species delimitation. A. COI. B. COI. C. ITS2. D. Combined data set

**A. Species delimitation – COI fragment: ASAP results**

**Partition 1**

Score: 1

Proba: 9.999900e-06

Nb subsets with recursion:6 (without recursion: 3)

------------------------------------------------------------

*Orbiniella petersenae sensu stricto*[ 1 ] n: 12 ;id: NBAAV685-23 NBAAV655-23 NBAAV659-23 NBAAV649-23 NBAAV660-23 NBAAV654-23 NBAAV662-23 NBAAV666-23 NBAAV653-23 NBAAV661-23 NBAAV648-23 NBAAV667-23

Subset[ 2 ] n: 2 ;id: NBAAV674-23 NBAAV697-23

*Orbiniella griegi* sp. nov.[ 3 ] n: 17 ;id: NBAAV673-23 NBAAV696-23 NBAAV695-23 NBAAV676-23 NBAAV651-23 NBAAV677-23 NBAAV683-23 NBAAV652-23 NBAAV663-23 POLNB1250-14 POLNB1257-14 ICBA263-16 ICBA264-16 NBAAV675-23 NBAAV678-23 NBAAV692-23 NBAAV693-23

*Orbiniella mayhemi* sp. nov.[ 4 ] n: 3 ;id: NBAAV664-23 NBAAV665-23 NBAAV650-23

*Orbiniella* sp.[ 5 ] n: 1 ;id: NBAAV684-23

*Orbiniella plumisetosa*[ 6 ] n: 1 ;id: Orbiniella_plumisetosa

**Partition 2**

Score: 2.5

Proba: 1.159328e-03

Nb subsets with recursion:6 (without recursion: 5)

------------------------------------------------------------

*Orbiniella petersenae sensu stricto*[ 1 ] n: 12 ;id: NBAAV685-23 NBAAV655-23 NBAAV659-23 NBAAV649-23 NBAAV660-23 NBAAV654-23 NBAAV662-23 NBAAV666-23 NBAAV653-23 NBAAV661-23 NBAAV648-23 NBAAV667-23

*Orbiniella griegi* sp. nov.[ 2 ] n: 2 ;id: NBAAV674-23 NBAAV697-23

*Orbiniella griegi* sp. nov.[ 3 ] n: 17 ;id: NBAAV673-23 NBAAV696-23 NBAAV695-23 NBAAV676-23 NBAAV651-23 NBAAV677-23 NBAAV683-23 NBAAV652-23 NBAAV663-23 POLNB1250-14 POLNB1257-14 ICBA263-16 ICBA264-16 NBAAV675-23 NBAAV678-23 NBAAV692-23 NBAAV693-23

*Orbiniella mayhemi* sp. nov.[ 4 ] n: 3 ;id: NBAAV664-23 NBAAV665-23 NBAAV650-23

*Orbiniella* sp.[ 5 ] n: 1 ;id: NBAAV684-23

*Orbiniella plumisetosa*[ 6 ] n: 1 ;id: Orbiniella_plumisetosa

**B. Species delimitation – 16S fragment: ASAP results**

**Partition 1**

Score: 1

Proba: 1.899981e-04

Nb subsets with recursion:7 (without recursion: 6)

------------------------------------------------------------

*Orbiniella petersenae sensu stricto*[ 1 ] n: 11 ;id: NBAAV666-23 NBAAV667-23 NBAAV685-23 NBAAV659-23 NBAAV661-23 NBAAV649-23 NBAAV662-23 NBAAV660-23 NBAAV648-23 NBAAV653-23 NBAAV654-23

*Orbiniella* sp. 279 PB[ 2 ] n: 1 ;id: CCFZP281-19

*Orbiniella* sp. 49 PB[ 3 ] n: 3 ;id: CCFZP051-19 CCFZP073-19 CCFZP164-19

*Orbiniella mayhemi* sp. nov.[ 4 ] n: 3 ;id: NBAAV650-23 NBAAV665-23 NBAAV664-23

*Orbiniella griegi* sp. nov.[ 5 ] n: 12 ;id: NBAAV692-23 NBAAV697-23 POLNB1257-14 NBAAV693-23 NBAAV695-23 NBAAV696-23 NBAAV677-23 NBAAV663-23 NBAAV652-23 NBAAV686-23 NBAAV676-23 NBAAV651-23

*Orbiniella parapari* sp. nov.[ 6 ] n: 18 ;id: NBAAV689-23 NBAAV658-23 NBAAV670-23 NBAAV669-23 NBAAV671-23 NBAAV672-23 NBAAV691-23 NBAAV656-23 NBAAV680-23 NBAAV681-23 NBAAV679-23 NBAAV682-23 NBAAV657-23 NBAAV668-23 NBAAV688-23 NBAAV690-23 NBAAV694-23 NBAAV687-23

*Orbiniella plumisetosa*[ 7 ] n: 1 ;id: Orbiniella_plumisetosa

**Partition 2**

Score: 3.5

Proba: 5.489022e-01

Nb subsets with recursion:3 (without recursion: 2)

------------------------------------------------------------

*Orbiniella petersenae sensu stricto*[ 1 ] n: 11 ;id: NBAAV666-23 NBAAV667-23 NBAAV685-23 NBAAV659-23 NBAAV661-23 NBAAV649-23 NBAAV662-23 NBAAV660-23 NBAAV648-23 NBAAV653-23 NBAAV654-23

*Orbiniella* sp. 279 PB/ *Orbiniella* sp. 49 PB/ *Orbiniella mayhemi* sp. nov./ *Orbiniella griegi* sp. nov./ *Orbiniella parapari* sp. nov.[ 2 ] n: 37 ;id: CCFZP281-19 CCFZP051-19 CCFZP073-19 CCFZP164-19 NBAAV650-23 NBAAV665-23 NBAAV664-23 NBAAV692-23 NBAAV697-23 POLNB1257-14 NBAAV693-23 NBAAV695-23 NBAAV696-23 NBAAV677-23 NBAAV663-23 NBAAV652-23 NBAAV686-23 NBAAV676-23 NBAAV651-23 NBAAV689-23 NBAAV658-23 NBAAV670-23 NBAAV669-23 NBAAV671-23 NBAAV672-23 NBAAV691-23 NBAAV656-23 NBAAV680-23 NBAAV681-23 NBAAV679-23 NBAAV682-23 NBAAV657-23 NBAAV668-23 NBAAV688-23 NBAAV690-23 NBAAV694-23 NBAAV687-23

*Orbiniella plumisetosa*[ 3 ] n: 1 ;id: Orbiniella_plumisetosa

**C. Species delimitation – ITS2 fragment: ASAP results**

**Partition 1**

Score: 2

Proba: 4.216270e-03

Nb subsets with recursion: 4 (without recursion: 3)

------------------------------------------------------------

*Orbiniella petersenae sensu stricto*[ 1 ] n: 8 ;id: NBAAV661-23 NBAAV666-23 NBAAV653-23 NBAAV659-23 NBAAV685-23 NBAAV654-23 NBAAV660-23 NBAAV662-23

*Orbiniella griegi* sp. nov.[ 2 ] n: 5 ;id: NBAAV676-23 NBAAV663-23 NBAAV651-23 NBAAV652-23 NBAAV678-23

*Orbiniella* sp./*Orbiniella mayhemi* sp. nov.[ 3 ] n: 4 ;id: NBAAV684-23 NBAAV665-23 NBAAV650-23 NBAAV664-23

*Orbiniella parapari* sp. nov.[ 4 ] n: 12 ;id: NBAAV679-23 NBAAV681-23 NBAAV656-23 NBAAV657-23 NBAAV658-23 NBAAV671-23 NBAAV669-23 NBAAV668-23 NBAAV682-23 NBAAV670-23 NBAAV672-23 NBAAV680-23

**Partition 2**

Score: 2

Proba: 1.037924e-01

Nb subsets with recursion: 5 (without recursion: 4)

------------------------------------------------------------

*Orbiniella petersenae sensu stricto*[ 1 ] n: 8 ;id: NBAAV661-23 NBAAV666-23 NBAAV653-23 NBAAV659-23 NBAAV685-23 NBAAV654-23 NBAAV660-23 NBAAV662-23

*Orbiniella griegi* sp. nov.[ 2 ] n: 5 ;id: NBAAV676-23 NBAAV663-23 NBAAV651-23 NBAAV652-23 NBAAV678-23

*Orbiniella* sp.[ 3 ] n: 1 ;id: NBAAV684-23

*Orbiniella mayhemi* sp. nov.[ 4 ] n: 3 ;id: NBAAV665-23 NBAAV650-23 NBAAV664-23

*Orbiniella parapari* sp. nov.[ 5 ] n: 12 ;id: NBAAV679-23 NBAAV681-23 NBAAV656-23 NBAAV657-23 NBAAV658-23 NBAAV671-23 NBAAV669-23 NBAAV668-23 NBAAV682-23 NBAAV670-23 NBAAV672-23 NBAAV680-23
